# Spatial heterogeneity, frequency-dependent selection and polymorphism in host-parasite interactions

**DOI:** 10.1186/1471-2148-11-319

**Published:** 2011-11-01

**Authors:** Aurélien Tellier, James KM Brown

**Affiliations:** 1 Section of Evolutionary Biology, Biocenter, University of Munich, 82152 Planegg-Martinsried, Germany; 2 Department of Disease and Stress Biology, John Innes Centre, Colney, Norwich, NR4 7UH, UK

**Keywords:** coevolution, natural selection, metapopulation dynamics, gene-for-gene relationship, resistance, avirulence, boom-and-bust cycles, frequency-dependent selection

## Abstract

**Background:**

Genomic and pathology analysis has revealed enormous diversity in genes involved in disease, including those encoding host resistance and parasite effectors (also known in plant pathology as avirulence genes). It has been proposed that such variation may persist when an organism exists in a spatially structured metapopulation, following the geographic mosaic of coevolution. Here, we study gene-for-gene relationships governing the outcome of plant-parasite interactions in a spatially structured system and, in particular, investigate the population genetic processes which maintain balanced polymorphism in both species.

**Results:**

Following previous theory on the effect of heterogeneous environments on maintenance of polymorphism, we analysed a model with two demes in which the demes have different environments and are coupled by gene flow. Environmental variation is manifested by different coefficients of natural selection, the costs to the host of resistance and to the parasite of virulence, the cost to the host of being diseased and the cost to an avirulent parasite of unsuccessfully attacking a resistant host. We show that migration generates negative direct frequency-dependent selection, a condition for maintenance of stable polymorphism in each deme. Balanced polymorphism occurs preferentially if there is heterogeneity for costs of resistance and virulence alleles among populations and to a lesser extent if there is variation in the cost to the host of being diseased. We show that the four fitness costs control the natural frequency of oscillation of host resistance and parasite avirulence alleles. If demes have different costs, their frequencies of oscillation differ and when coupled by gene flow, there is amplitude death of the oscillations in each deme. Numerical simulations show that for a multiple deme island model, costs of resistance and virulence need not to be present in each deme for stable polymorphism to occur.

**Conclusions:**

Our theoretical results confirm the importance of empirical studies for measuring the environmental heterogeneity for genetic costs of resistance and virulence alleles. We suggest that such studies should be developed to investigate the generality of this mechanism for the long-term maintenance of genetic diversity at host and parasite genes.

## Background

Disease is a major driving force of evolution, generating natural selection which acts both on host defences and on genes enabling parasites to overcome those defences. Two types of polymorphism have been proposed to result from the co-evolution of interacting host and parasite loci [1]. Long-term maintenance of polymorphism is predicted by the "trench warfare" hypothesis, resulting from balancing selection acting on both host and parasite genes [1,2]. Polymorphism at host and parasite loci is thus predicted to be ancient [2] with substantial phenotypic and molecular diversity within species at population and metapopulation levels. Alternatively, in the "arms race" scenario, there is recurrent fixation of favourable alleles by selective sweeps. In this situation, alleles are short-lived, and transient polymorphism is only observable for a short period of time [1]. An important topic in population genetics of host-parasite interactions is to understand the ways in which natural selection interacts with the organisms' ecology to promote the occurrence of each scenario in natural populations [3]. The occurrence of "arms race" or "trench warfare" dynamics has been studied in controlled laboratory experiments with bacterium-phage systems and theoretical predictions have been tested [4-10].

The gene-for-gene (GFG) relationship, found widely in plant diseases, but also in some invertebrate-parasite systems [11,12], is a model system for host-parasite co-evolution because the molecular biology of the interactions between host and parasite genotypes is well-understood [13]. In the GFG system a host can resist attack by a parasite if it has a resistance (*RES*) gene which enables recognition of a specific parasite avirulence (*AVR*) gene. A parasite is not detected by a host and resistance is ineffective if the host has a susceptibility allele (*res*) or the parasite has a virulence allele (*avr*). Coevolutionary dynamics are driven by indirect frequency-dependent selection (iFDS), in which the strength of natural selection acting on resistance genes depends on the frequencies of parasite genes and *vice-versa *[14,15]. The resulting "boom-and-bust" behaviour causes unstable coevolutionary cycles, leading to fixation of alleles in host and parasite populations (an "arms race" scenario). In this case, there is a point at which there is balanced polymorphism at the host *RES *and parasite *AVR *loci but this equilibrium is unstable [16]. Long term maintenance of polymorphism in GFG systems, *i.e. *via stable equilibrium or cycling of host and parasite allele frequencies, generally requires the existence of a stable equilibrium point at which there is balanced polymorphism at *RES *and *AVR *loci. Conditions for such a stable equilibrium have been the subject of theoretical population genetics studies which have emphasised the existence of genetic fitness costs associated with resistance and virulence alleles [17-20], prompting attempts to estimate such costs experimentally [21-25].

However, an important recent theoretical result is that direct frequency-dependent selection (FDS), in which the contribution of an allele to fitness depends on its own frequency, is necessary for balanced polymorphism to be maintained [15]. Specifically, polymorphism can be stabilised if direct FDS is negative, so that the allele's net contribution to fitness declining with increasing frequency. Diverse epidemiological and ecological processes which act within a single population generate negative direct FDS and stabilise polymorphism [3,15,26,27]. Crucially, while fitness costs of resistance and virulence alleles are required to generate coevolutionary cycles, they do not generate direct FDS [15]. In other words, a single-locus GFG model, in which each host and parasite locus has two alleles, with discrete non-overlapping generations of haploid parasites and haploid or selfing plants, generates an "arms race" with recurrent fixation of host and parasite alleles, if the life cycle of host and parasites are synchronised and disease transmission is frequency-dependent [15,28].

Most organisms exhibit some degree of spatial structuring of their populations, which has two main influences on the dynamics of host-parasite systems. Firstly, metapopulation structure, in which a species exists as a set of local host and parasite demes, generates demographic processes such as random genetic drift, limited dispersal between demes and extinction-recolonisation events, driving local adaptation of hosts or parasites [29]. However, metapopulation dynamics within a homogeneous environment, in which coevolutionary parameters are identical in all demes, do not in themselves stabilise a model which would otherwise be unstable in a single population [30]. Secondly, the ranges of most species extend over diverse ecological habitats which generate different rates of natural selection, driving local adaptation of populations to abiotic and biotic environments [31]. Since the seminal work of Levene [32], much theoretical work has focused on the mathematical conditions for maintenance of polymorphism for a diploid species in two (or more) niches linked by gene flow with different selective coefficients for different alleles [33-36]. Briefly, a heterogeneous environment with different strengths of natural selection in different demes and weak coupling between demes, *i.e. *low level of gene flow, favour stable local and global polymorphic equilibria by migration-selection balance (review in [37,38]) or heterozygous advantage [36].

A general theory which summarises the dynamics of coevolution in a spatially heterogeneous environment is the 'geographic mosaic of co-evolution'. This proposes that variation in selection pressures between demes is caused by heterogeneous abiotic conditions generating 'hot' and 'cold' spots of coevolution [39]. Gavrilets and Michalakis [30] analysed a general model for coevolution in heterogeneous environment. Polymorphism may occur in a multilocus GFG system in a metapopulation where heterogeneous selection is generated by hot and cold (no parasite present) spots [40]. Theoretical and experimental studies considered heterogeneity in space for the cost for the host of being diseased or absence of parasite [30,40,41]. In this paper, we bring together the theory of host-parasite coevolution in single populations with that of heterogeneous environments, and investigate the conditions which lead to balanced polymorphism at host and parasite loci both in individual demes and in the metapopulation as a whole. This study has four objectives.

First, we connect the existing theory [33,34,37,38] to host-parasite coevolutionary models [15], by showing that migration-selection dynamics generate negative direct FDS.

Second, we show that in GFG models, negative direct FDS arising from gene flow between demes with different environments can stabilise polymorphism at interacting host and parasite loci.

Third, we extend previous work [30,40] to relate the strength of direct FDS and the conditions for occurrence of stable polymorphism to the parameters which describe plant-parasite interactions involving GFG relationships. In particular, we highlight the importance of costs of resistance and virulence for the metapopulation dynamics of GFG coevolution, and show that these costs are not needed in every deme for polymorphism to be stable.

Fourth, we discuss the relevance and implications of our results for empirical studies. We recommend testing for the existence of genetic costs for resistance and virulence alleles and measuring their environmental variability in crop and natural plant-parasite systems [22-25], and their influence on coevolutionary dynamics in bacteria-phage systems [5-10,42].

### General model of selection in heterogeneous habitats

#### Model description

We consider a metapopulation divided into *n *demes connected by migration in which a gene has two alleles, *G *and *g*, with frequencies *G_i _*and *g_i _*respectively in deme *i *(*G_i _*+ *g_i _*= 1). The species in the model is either haploid or, if diploid, is selfing with no remaining heterozygosity at the locus in question. We assume that soft selection occurs because plants compete for resources locally and thus the number of individuals per deme is fixed (and very large) [43]; specifically, we assume for convenience that all demes have infinite population size. Each generation takes place as a two step process: 1) natural selection occurs within each deme, altering allele frequencies, then 2) migration occurs between demes (similar results when migration precedes selection are shown in the Additional File 1, section 2). In deme *i*, allele *G *has a fitness of (1 - *τ_i_*) and allele *g *a fitness of (1 - *σ_i_*). After natural selection has occured in deme *i*, the frequencies of the two alleles are:

$$G_{i}^{\prime }=\frac{G_{i}\left(1-\tau_{i}\right)}{1-G_{i}\tau_{i}-g_{i}\sigma_{i}}$$

and

(1) $$g_{i}^{\prime }=\frac{g_{i}\left(1-\sigma_{i}\right)}{1-G_{i}\tau_{i}-g_{i}\sigma_{i}}$$

where *G_i_*' is the frequency of *G *in deme *i *after natural selection (with *G_i_*' + *g_i_*' = 1).

Throughout this paper, we analyse ratios of allele frequencies to calculate frequency changes between generations. This has two advantages over the more usual analysis of frequencies of single alleles (use of forward and backward migration matrices [38]). Firstly, it dispenses with complicated terms for mean fitness, greatly simplifying differentiation of functions of allele frequencies. Secondly, in the absence of direct FDS, the rate of change of the logarithm of the ratio of the frequencies of two alleles at a locus (the logit of the frequency used as the numerator) is constant if the strength of natural selection is independent of allele frequencies. A non-zero first derivative of the rate of change of the logit-frequency therefore implies the existence of direct FDS.

After natural selection has occurred, there is a migration phase during which deme *i *receives alleles from other demes (*j*). *G_i_*″ and *g_i_*″ are then the frequencies of the two alleles in deme *i *after the migration step (*G_i_*″ + *g_i_*″ = 1), At the end of the generation under consideration:

(2) $$\frac{G_{i}^{\prime \prime }}{g_{i}^{\prime \prime }}=\frac{{G_{i}}^{\prime }L_{i}+\underset{j\neq i}{\sum }{G_{j}}^{\prime }m_{ji}}{g_{i}^{\prime }L_{i}+\underset{j\neq i}{\sum }{g_{j}}^{\prime }m_{ji}}$$

where *m_ji _*is the proportion of the population in deme *i *which originated in deme *j *(i.e. the migration rate from deme *j *to *i*) and $L_{i}=1-\underset{j\neq i}{\sum }m_{ji}$ is the proportion of the population in deme *i *which originated in deme *i *itself. We assume that the allele of interest does not affect the probability of migration.

If there is no direct FDS, the strength of natural selection on an allele does not depend on its frequency, so the change in log(*G_i_*/*g*_i_) (the logit transformation of *G_i_*) over a generation is independent of *G_i_*. If *γ_i _= *log(*G_i_*/*g_i_*) and Δ*γ_i _*= *γ_i_*″-*γ_i_*, the change in *γ_i _*between generations, then for given values of allele frequencies *G_j _*and *g_j _*in demes *j *(*j *≠ *i*),

(3) $$\text{Δ}\gamma_{i}=\log\left\{\frac{\left(1-\tau_{i}\right)L_{i}+\varphi_{i}\left[\sigma_{i}-\tau_{i}+{G_{i}}^{-1}\left(1-\sigma_{i}\right)\right]}{\left(1-\sigma_{i}\right)L_{i}+\psi_{i}\left[\tau_{i}-\sigma_{i}+{g_{i}}^{-1}\left(1-\tau_{i}\right)\right]}\right\}$$

where *φ_i _*and *ψ_i _*are constants for given values of *G_j _*and *g_j _*and depend on the migration rates (*m_ji_*) and the selection coefficients in deme *j *(*τ_j _*and *σ_j_*):

$$\varphi_{i}=\underset{j\neq i}{\sum }\frac{G_{j}\left(1-\tau_{j}\right)m_{ji}}{1-G_{j}\tau_{j}-g_{j}\sigma_{j}}$$

and

$$\psi_{i}=\underset{j\neq i}{\sum }\frac{g_{j}\left(1-\sigma_{j}\right)m_{ji}}{1-G_{j}\tau_{j}-g_{j}\sigma_{j}}$$

From (3), *d*Δ*γ_i_*/*dγ_i _*< 0 when there is migration (*L_i _*< 1 ⇔ *m_ji _*> 0) and *σ*, *τ *or both differ between at least some demes (details of calculations in Additional File 1, section 1). Hence, there is negative direct FDS, with the net benefit of allele *G *to fitness declining (or its net cost increasing) as its frequency increases. *d*Δ*γ_i_*/*dγ_i _*is never positive, so the selective advantage of an allele never increases with increasing frequency.

The conditions for metapopulation structure to generate negative direct FDS in deme *i *(*d*Δ*γ_i_*/*dγ_i _*< 0) are therefore that the selection coefficients (*σ_i _*and *τ_i_*) on the alleles differ between deme *i *and one or more other demes (*σ_j _*and *τ_j_*) and that there is gene flow (for example, by migration) from other demes into deme *i *(*m_ji _*> 0 for at least some *j*). In biological terms, negative direct FDS is generated by heterogeneous environments in a metapopulation because as *G_i _*increases, the net loss of *G *alleles from population *i *to other populations increases. Hence the net rate of selection for *G *(or against *g*) in deme *i *falls as *G_i _*rises. This is the process described elsewhere as direct FDS [15]. These are necessary but not sufficient conditions for stable polymorphism, because an additional condition is that the two alleles must have identical mean fitnesses at some value of *G_i_*/*g_i _*to generate balanced polymorphism due to migration-selection balance [33,38]. Note also that negative direct FDS is also generated if migration precedes selection ([38]; Additional File 1, section 2).

### Gene-for-gene model in heterogeneous habitats

#### Methods

##### Model description

Both hosts and parasites are haploid or selfing with no heterozygotes at the loci of interest and the population is of infinite size with a soft selection model. There are *n *demes. Infection is monocyclic, with each plant attacked once by one parasite in each generation [15,17]. In this simple GFG system, a plant has one locus with two alleles, resistant (*RES*) and susceptible (*res*), and the parasite one pathogenicity locus with two alleles, avirulent (*AVR*) and virulent (*avr*). A parasite is not detected by a host and resistance is ineffective if the host has a susceptibility allele (*res*) or the parasite has a virulence allele (*avr*); in this case the fractional reduction in a plant's reproductive fitness being diseased is *s_i _*in deme *i *(Table 1). When the plant has a *RES *allele matching the *AVR *allele of the parasite by which it is attacked, the plant mounts a successful resistance reaction, preventing the parasite from causing disease. The plant fitness is thus 1, and the fitness of *AVR *parasites attacking *RES *plants is 1-*c_i_*. Normally, *c_i _*≈ 1 as these attacks are usually unsuccessful (Table 1), but note that *c_i _*< 1 leads to a model of partial resistance [44]. The *RES *and *avr *alleles have constitutive fitness costs *u_i _*and *b_i _*to hosts and parasites respectively in deme *i *[15,17]. In the metapopulation, GFG co-evolution takes place in a spatial structure in which demes are linked by migration of host seeds or pollen and of parasite spores. As in (2), selection in each deme takes place before migration between demes. We use models of gene frequencies, typical of population genetics, to investigate long-term outcomes of coevolution over time-scales of hundreds or thousands of generations; this means that epidemiological and life-cycle parameters are not specified explicitly but absorbed in the four fitness costs. Our model therefore implies that, as in most previous theoretical research on the GFG system, epidemiological processes can be regarded as density-independent.

**Table 1:** Fitnesses of hosts and parasites in deme number *i*.

		Fitness	
		
Host genotypes (frequencies)	Parasite genotypes (frequencies)	Parasite	Host
*RES *(*R_i_*)	*AVR *(*A_i_*)	1-*c_i _*	1-*u_i_*
	
	*avr *(*a_i_*)	1-*b_i_*	(1-*u_i_*)(1-*s_i_*)

*res *(*r_i_*)	*AVR *(*A_i_*)	1	1-*s_i_*
	
	*avr *(*a_i_*)	1-*b_i_*	1-*s_i_*

All parameters are in the range 0 to 1.

Extending the notation of (1-3), the subscripts *H *and *P *refer to the host and parasite. *R_i _*is the current frequency of *RES *resistance alleles in deme *i *(respectively, *r_i _*is the frequency of *res *susceptibility alleles), and *R_i_*' (*r_i_*') the frequency in the next generation. Similarly *A_i _*and *a_i _*are the frequencies of avirulent (*AVR*) and virulent (*avr*) parasites. The recurrence equation for the change in the ratio of *RES *to *res *hosts in deme *i *between generations is calculated from Table 1:

$$\frac{{R_{i}}^{\prime }}{{r_{i}}^{\prime }}=\frac{R_{i}\left(1-u_{i}\right)\left(1-s_{i}a_{i}\right)L_{Hi}}{r_{i}\left(1-s_{i}\right)L_{Hi}+\underset{j\neq i}{\sum }r_{j}\left(1-s_{j}\right)m_{Hji}}$$

(4) $$+\frac{\underset{j\neq i}{\sum }R_{j}\left(1-u_{j}\right)\left(1-s_{j}a_{j}\right)m_{Hji}}{r_{i}\left(1-s_{i}\right)L_{Hi}+\underset{j\neq i}{\sum }r_{j}\left(1-s_{j}\right)m_{Hji}}$$

Similarly, in the parasite population, the recurrence equation for the ratio of *AVR *to *avr *frequencies in deme *i *is:

$$\frac{{A_{i}}^{\prime }}{{a_{i}}^{\prime }}=\frac{A_{i}\left(1-c_{i}R_{i}\right)L_{Pi}}{a_{i}\left(1-b_{i}\right)L_{Pi}+\underset{j\neq i}{\sum }a_{j}\left(1-b_{j}\right)m_{Pji}}$$

(5) $$+\frac{\underset{j\neq i}{\sum }A_{j}\left(1-c_{j}R_{j}\right)m_{Pji}}{a_{i}\left(1-b_{i}\right)L_{Pi}+\underset{j\neq i}{\sum }a_{j}\left(1-b_{j}\right)m_{Pji}}$$

In (4-5), host and parasite allele frequencies at generation *t*+1 are composed of a fraction *L_Hi _*(*L_Pi_*) from deme *i*, and the sum of fractions *m_Hji _*(*m_Pji_*) of migrants from all other demes *j ≠ i *to deme *i*. There are trivial equilibrium points where one allele is fixed in each species throughout the metapopulation but there is also a non-trivial, interior equilibrium where $R_{i}=R_{i}^{\prime }={\hat{R}}_{i}$ and $A_{i}=A_{i}^{\prime }={Â}_{i}$. Values of the equilibrium frequencies of host alleles when the parasite (but not the host) migrates between demes, or *vice-versa*, are derived in the Additional File 1, section 3. Equilibrium frequencies in the parasite population depends on the host costs, *u *and *s *[14], and also on the host migration parameters (*L_H _*and *m_H_*). Similarly, host allele equilibrium frequencies depend on the parasite fitness parameters *b *and *c*, as well as on parasite migration rates (*L_P _*and *m_P_*).

In the special case when there is no migration in either species, the dynamics of the GFG system is described by a pair of recurrence equations for a GFG model in a single population [15,27]:

$$\frac{R_{i}^{\prime }}{{r^{\prime }}_{i}}=\frac{R_{i}}{r_{i}}\cdot \frac{\left(1-u_{i}\right)\left(1-s_{i}a_{i}\right)}{1-s_{i}}$$

and

(6) $$\frac{A_{i}^{\prime }}{a_{i}^{\prime }}=\frac{A_{i}}{a_{i}}\cdot \frac{1-c_{i}R_{i}}{1-b_{i}}$$

In addition to trivial equilibrium points, the non-trivial, interior equilibrium is defined by $R_{i}=R_{i}^{\prime }={\hat{R}}_{i}$ and $A_{i}=A_{i}^{\prime }={Â}_{i}$:

$${\hat{R}}_{i}=\frac{b_{i}}{c_{i}}$$

and

(7) $${Â}_{i}=\frac{u_{i}\left(1-s_{i}\right)}{s_{i}\left(1-u_{i}\right)}$$

This equilibrium point is unstable in a population which is not connected to any other population, because there is only indirect FDS, and not negative direct FDS in (6). Any deviation of gene frequencies from equilibrium ultimately results in fixation of host and parasite alleles [15].

##### Model description with *2 *demes

For further analysis of the GFG coevolutionary model, we simplify the system of equations (4-5) into a model of two demes linked by migration. We assume symmetrical migration between the two demes (following [30]), so that *m_Pij _*= *m_Pji _*= *m_P _*and *m_Hij _*= *m_Hji _*= *m_H_*. Host (*m_H_*) and parasite (*m_P_*) migration rates can be chosen with equal or different values. The cost of resistance, cost of virulence, cost of being diseased and cost to an *AVR *parasite of infecting a *RES *plant are respectively *u_1_, b_1_, s_1 _*and *c_1 _*in deme 1 and *u_2_, b_2_, s_2 _*and *c_2 _*in deme 2. The system of equations (4, 5) becomes as follows for deme 1:

(8) $$\frac{R_{1}\prime }{r_{1}\prime }=\frac{\left(1-m_{H}\right)R_{1}\left(1-u_{1}\right)\left(1-s_{1}a_{1}\right)+m_{H}R_{2}\left(1-u_{2}\right)\left(1-s_{2}a_{2}\right)}{\left(1-m_{H}\right)r_{1}\left(1-s_{1}\right)+\;m_{H}r_{2}\left(1-s_{2}\right)}$$

and

(9) $$\frac{A_{1}\prime }{a_{1}\prime }=\frac{\left(1-m_{p}\right)A_{1}\left(1-c_{1}R_{1}\right)+m_{p}A_{2}\left(1-c_{2}R_{2}\right)}{\left(1-m_{p}\right)a_{1}\left(1-b_{1}\right)+\;m_{p}a_{2}\left(1-b_{2}\right)}$$

Without migration, *m_P _*= *m_H _*= 0, this system collapses to the GFG system analysed in [15], which has no stable equilibrium at which the host and parasite are polymorphic.

## Results

### Analytical results for a GFG model with *n *demes

We first demonstrate that gene flow between heterogeneous demes generates negative direct FDS on both *RES *and *AVR *loci. For simplicity of notation from (4, 5), the frequencies ${\tilde{R}}_{i}$ and ${\tilde{r}}_{i}$ of the *RES *(and *res*) alleles after selection and before migration are ${\tilde{R}}_{i}=R_{i}\left(1-u_{i}\right)\left(1-s_{i}a_{i}\right)$ and ${\tilde{r}}_{i}=r_{i}\left(1-s_{i}\right)$. The change in the log-ratio of the frequencies *R_i _*and *r_i _*at the end of generation *t*, *ρ_i _*= log(*R_i_*/*r_i_*), is

$$\text{Δ}\rho_{i}=\log\left({\tilde{R}}_{i}L_{Hi}+\underset{j\neq i}{\sum }{\tilde{R}}_{j}m_{Hji}\right)$$

$$-\log\left({\tilde{r}}_{i}L_{Hi}+\underset{j\neq i}{\sum }{\tilde{r}}_{j}m_{Hji}\right)-\rho_{i}$$

so the rate of selection on resistance in the host population is (Additional File 1, sections 3, 4):

$$\frac{d\text{Δ}\rho_{i}}{d\rho_{i}}=-\frac{{\tilde{R}}_{i}R_{i}L_{Hi}\underset{j\neq i}{\sum }{\tilde{r}}_{j}m_{Hji}+{\tilde{r}}_{i}r_{i}L_{Hi}\underset{j\neq i}{\sum }{\tilde{R}}_{j}m_{Hji}}{\left({\tilde{R}}_{i}L_{Hi}+\underset{j\neq i}{\sum }{\tilde{R}}_{j}m_{Hji}\right)\left({\tilde{r}}_{i}L_{Hi}+\underset{j\neq i}{\sum }{\tilde{r}}_{j}m_{Hji}\right)}$$

(10) $$+\frac{\left(\underset{j\neq i}{\sum }{\tilde{R}}_{j}m_{Hji}\right)\left(\underset{j\neq i}{\sum }{\tilde{r}}_{j}m_{Hji}\right)}{\left({\tilde{R}}_{i}L_{Hi}+\underset{j\neq i}{\sum }{\tilde{R}}_{j}m_{Hji}\right)\left({\tilde{r}}_{i}L_{Hi}+\underset{j\neq i}{\sum }{\tilde{r}}_{j}m_{Hji}\right)}$$

This differential is never positive. It is always negative, especially when gene frequencies are close to the equilibrium point, implying that there is negative direct FDS on the *RES *gene if there is migration between population *i *and other populations and if the fitness cost of resistance (*u*) or the cost of disease (*s*) differs between at least some populations, i.e. for some demes *j*, *u_i _≠ u_j _*(or *s_i _≠ s_j_*). Note that these results do not assume any specific pattern of migration in the metapopulation.

Similarly for the parasite frequencies, writing ${Ã}_{i}=A_{i}\left(1-c_{i}R_{i}\right)$ and ${ã}_{i}=a_{i}\left(1-b_{i}\right)$, the change in *α_i _*= log(*A_i_*/*a_i_*) is

$$\text{Δ}\alpha_{i}=\log\left({Ã}_{i}L_{Pi}+\underset{j\neq i}{\sum }{Ã}_{j}m_{Pji}\right)$$

$$-\log\left({ã}_{i}L_{Pi}+\underset{j\neq i}{\sum }{ã}_{j}m_{Pji}\right)-\alpha_{i}$$

The equation that describes direct FDS on the *AVR *locus is thus:

(11) $$\frac{d\text{Δ}\alpha_{i}}{d\alpha_{i}}=-1+A_{i}a_{i}\left(\frac{\left(1-c_{i}R_{i}\right)L_{Pi}}{{Ã}_{i}L_{Pi}+\underset{j\neq i}{\sum }{Ã}_{j}m_{Pji}}\right)\left(+\frac{\left(1-b_{i}\right)L_{Pi}}{{ã}_{i}L_{Pi}+\underset{j\neq i}{\sum }{ã}_{j}m_{Pji}}\right)$$

This differential is never positive, implying that negative direct FDS on the *AVR *gene is generated by migration of the parasite between populations in which the cost of virulence (*b*) or the cost to being unable to infect a *RES *host (*c*) varies between at least some populations (Additional File 1, sections 3-4), i.e. for some demes *j*, *b_i _≠ b_j _*(or *c_i _≠ c_j_*).

We are interested here in the stability of a local equilibrium point (in a given deme *i*), which is given by the eigenvalues of the Jacobian matrix *J_i _*for the given dynamical system (4-5).

(12) $$J_{i}=\left(\begin{array}{cc} \frac{d\text{Δ}\rho_{i}}{d\rho_{i}} & \frac{d\text{Δ}\rho_{i}}{d\alpha_{i}}\\ \frac{d\text{Δ}\alpha_{i}}{d\rho_{i}} & \frac{d\text{Δ}\alpha_{i}}{d\alpha_{i}} \end{array}\right)$$

For the polymorphic equilibrium to be stable in a discrete time dynamical system, both eigenvalues of *J_i _*must lie within a unit circle centered on (-1, 0) in the complex plane. In other words, a necessary condition for stability is that the trace of the Jacobian matrix (8-9; the sum of the diagonal elements) is negative. This is the case if *dΔρ_i_*/*dρ_i _+ dΔα_i_*/*dα_i _< 0 *[15]. This condition implies that there is negative direct FDS at either the *RES *locus, the *AVR *locus or both, because the change in *R_i _*depends on its own value as well as on *a_i _*and *vice-versa *(10-11).

### Analytical results for a GFG model with two demes

Having shown in (8, 9) that direct FDS is generated in this GFG coevolutionary model with *n *demes, we now investigate which coevolutionary parameters are most important for promoting stable, balanced polymorphism in a simplified GFG system with 2 demes (8, 9). In the following analysis, the frequency *θ_i _*of coevolutionary cycles is calculated close to the internal equilibrium point in a single deme *i *(assuming no migration). This can be computed approximately by linearising the system of equations around the interior equilibrium point. Without migration, in deme *i*,

(13) $$\theta_{i}\approx \frac{1}{2\pi }\arccos{\left\{1+\frac{b_{i}\left(c_{i}-b_{i}\right)\left(s_{i}-u_{i}\right)u_{i}}{\left(1-b_{i}\right)c_{i}s_{i}\left(1-u_{i}\right)}\right\}}^{-0.5}$$

(Additional File 1, section 6). *θ_i _*depends strongly on the values of *b_i _*and *u_i_*, either of which increase *θ_i_*. The frequency of cycles depends more weakly on *s_i_*, increasing with *s_i _*especially when *s_i _*is not much greater than *u_i_*. In the usual case when *c_i _*is close to 1 (*i.e*. when AVR parasites have very low fitness on RES plants) variation in *c_i _*does not affect *θ_i _*greatly.

In our GFG system with two demes, the difference between the frequencies of oscillation of the coevolutionary cycles in the two demes is proportional to (*c*_1 _= *c*_2 _= 1):

(14) $$\text{Δ}\theta \propto |\frac{b_{1}u_{1}\left(s_{1}-u_{1}\right)}{s_{1}\left(1-u_{1}\right)}-\frac{b_{2}u_{2}\left(s_{2}-u_{2}\right)}{s_{2}\left(1-u_{2}\right)}|$$

We can then calculate the diagonal coefficients of the Jacobian matrix *J_1 _*in deme 1 as a function of the equilibrium allele frequencies and the difference between the oscillation frequencies (Eq. S7.10, Additional File 1, section 7). We assume *b_2 _*= *b *and *b_1 _*= *b *+ *β *but equal costs *u *and *s *between demes (*u_1 _*= *u_2 _*= *u *and *s_1 _*= *s_2 _*= *s*):

(15) $$\frac{d\text{Δ}\rho_{1}}{d\rho_{1}}\approx \frac{-b\left(1-b\right)uâm_{H}}{4\pi \text{Δ}\theta \left(1-2b\right)\left(1-m_{H}\right)+b\left(1-b\right)uâ}$$

This implies that as the natural oscillation frequencies in the two demes diverge (Δ*θ *increases as *β *increases) because the costs of virulence *b_1 _*and *b_2 _*differ between demes, the strength of direct FDS on *R *changes in the two demes. With increasing Δ*θ *(13, 15), direct FDS thus becomes more negative in the deme with the lower cost *b *(here deme 2), and less negative in the deme with the higher cost *b *(here deme 1).

Now assuming different costs of virulence *u *(*u_2 _*= *u *and *u_1 _*= *u *+ *ε*) or of disease *s *(*s_1 _*= *s *and *s_2 _*= *s *+ *σ*) but equal *b *(*b_1 _*= *b_2 _*= *b*) between demes:

(16) $$\frac{d\text{Δ}\alpha_{1}}{d\alpha_{1}}\approx \frac{-buÂâm_{P}}{4\pi \text{Δ}\theta \left(â-Â\right)\left(1-m_{H}\right)+buÂâ}$$

As *s *or *u *diverges between demes, Δ*θ *increases (because *ε *and *σ *increase) so direct FDS alters, with *d*Δ*α*_2_/*dα*_2 _becoming more negative in the deme with lower *u *or higher *s *(here deme 2). Conversely, *d*Δ*α*_1_/*dα*_1 _becomes less negative in the deme with the higher *u *or lower *s *(here deme 1).

Equations (14, 15) demonstrate a close relationship between Δ*θ *and direct FDS. This can be analysed close to the interior equilibrium point when the host cost parameters, *s *and *u*, are the same in both demes and there is no gene flow in the pathogen, or when the cost of pathogen virulence, *b*, is constant and there is no host gene flow. In the former case, Δ*θ *increases and *dΔα_i_*/*dα_i_*, becomes more negative as *b *diverges between demes (14). In the latter case, Δ*θ *increases and *dΔρ_i_*/*dρ_i _*becomes more negative as the cost of being diseased, *s *diverges between demes, and also as *u *diverges so long as *u_i _*≲ *s*/2 in deme *i *(see (13), Additional File 1, section 7).

### Simulation results for 2 demes

We explore the behaviour of the GFG model with two demes (8, 9) with respect to the various coevolutionary parameters in order to determine the parameters which most strongly influence the coevolutionary dynamics. Analytical conditions for the non-trivial equilibrium point to be stable cannot be derived because the dynamics of (4-5) are non-linear and there is no general solution for (*R_i_, a_i_*) in a closed form. The quantitative behaviour of the system in general was therefore studied using numerical simulations.

**Figure 1:**
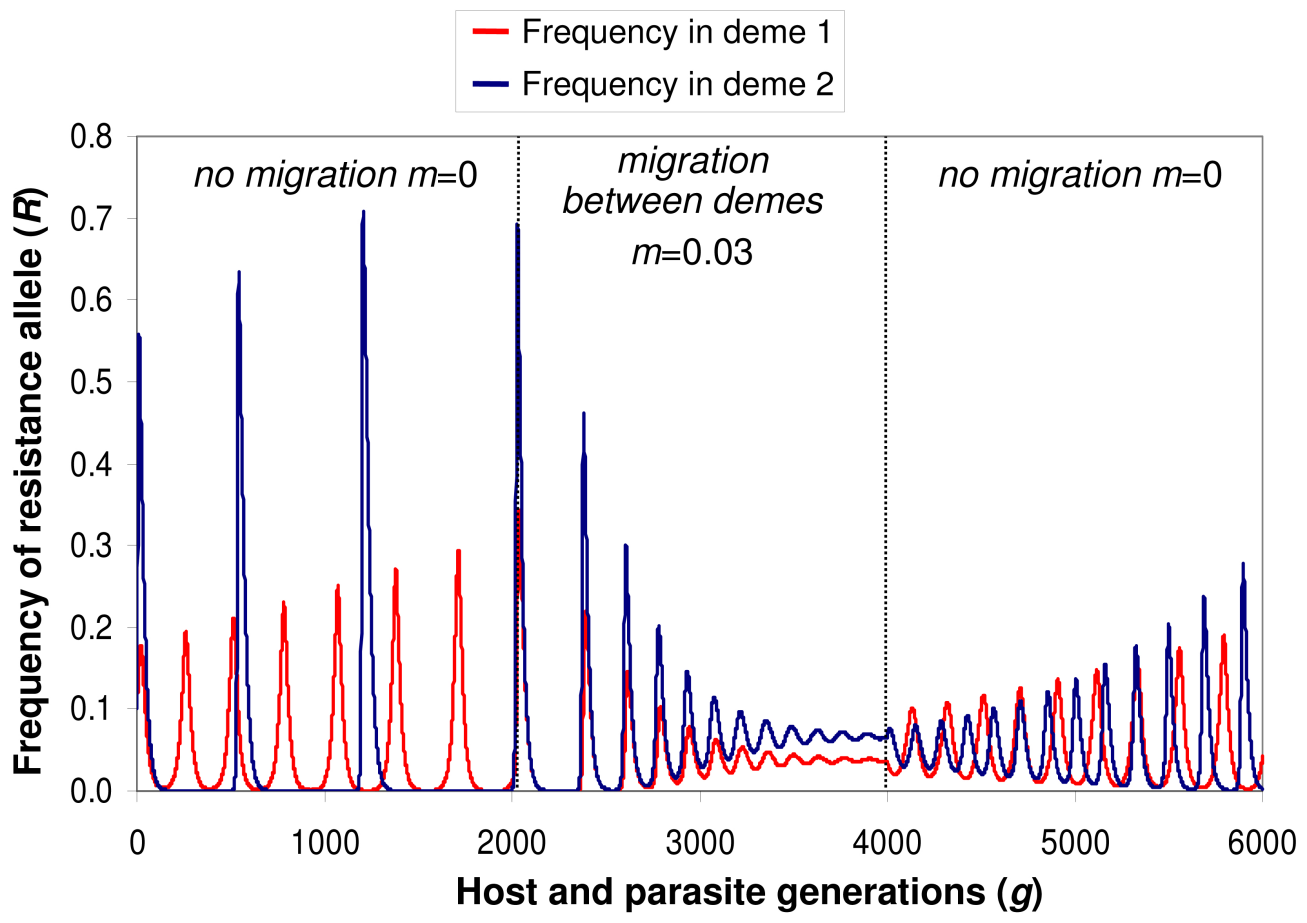
**Dynamics of the frequencies of a resistance (*RES*) allele in two demes linked by migration**. The model is of a gene-for-gene interaction between a host *RES *gene and a parasite avirulence (*AVR*) gene. The costs to the host of having the *RES *allele (*u*) or the parasite the virulence (*avr*) allele (*b*) are 0.05 in both demes. The cost to a plant of being diseased (*s*) is 0.1 in deme 1 (red) and 0.3 in deme 2 (blue). First, the model was run without migration between the demes for 1000 generations; the oscillations in the two demes had different frequencies and spiralled outwards from the interior equilibrium point. After 2000 generations, migration was introduced with a fraction 0.03 of the population being dispersed between the two demes; the oscillations in the two demes became synchronised and damped one another, thus stabilising polymorphism. After a further 2000 generations, migration was eliminated again, resulting once again in expanding, asynchronous oscillations in the two demes.

**Figure 2:**
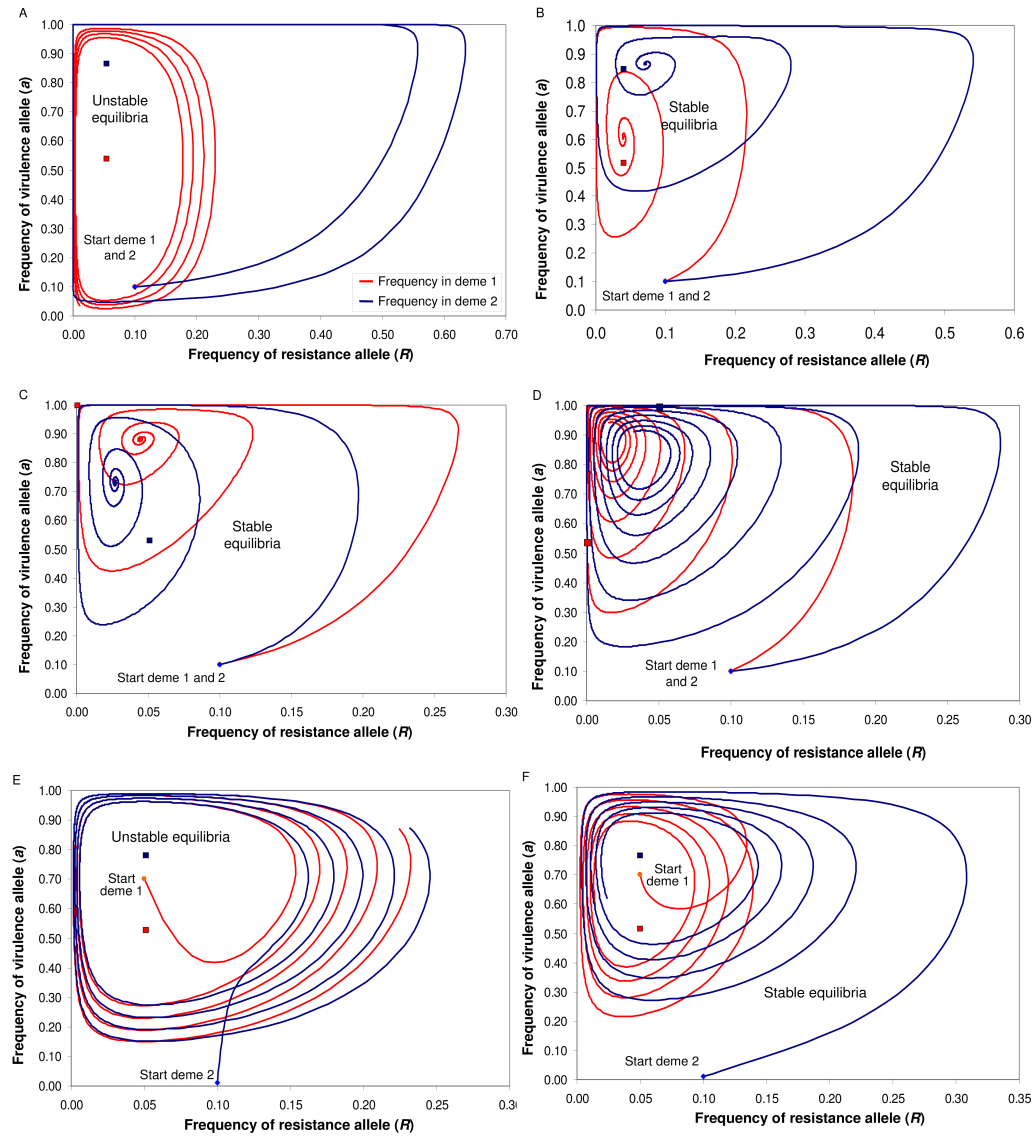
**Dynamics of allele frequencies in a two-deme model with unequal parameter values between demes**. The positions of the calculated equilibria are shown for single populations with no migration (red and blue rectangle for deme 1 and 2; for simplicity, *c *= 1). a) Different costs of disease, no migration (*u_1 _*= *u_2 _*= 0.05, *b_1 _*= *b_2 _*= 0.05, *s_1 _*= 0.1, *s_2 _*= 0.3, *m *= 0): unstable dynamics, as the graph of (*R*,*a*) spirals outwards with different frequencies in each deme. b) Different costs of disease, with migration (*u_1 _*= *u_2 _*= 0.05, *b_1 _*= *b_2 _*= 0.05, *s_1 _*= 0.1, *s_2 _*= 0.3, *m *= 0.03): stable dynamics, with synchronised oscillations in the two demes spiralling inwards towards the interior equilibrium points. c) Fitness costs of *RES *and *avr *in one deme but not the other (*b_2 _*= *u_2 _*= 0.05, *b_1 _= u_1 _*= 0, *s_1 _*= *s_2 _*= 0.1, *m *= 0.03): synchronised, stabilising oscillations. d) No cost of *RES *in one deme, no cost of *avr *in the other (*b_1 _*= *u_2 _*= 0, *b_2 _= u_1 _*= 0.05, *s_1 _*= *s_2 _*= 0.1, *m *= 0.03): synchronised, stabilising oscillations. e) Identical costs of resistance and virulence but different costs of disease (*b_1 _*= *b_2 _*= 0.05, *u_1 _= u_2 _*= 0.05, *s_1 _*= 0.1, *s_2 _*= 0.2), initial allele frequencies are (*R*, *a*) = (0.05, 0.7) in deme 1 and (0.1, 0.01) in deme 2: unstable dynamics occurs if migration *m *= 0.2. f) Identical parameters as in (e), initial allele frequencies are (0.05, 0.7) in deme 1 and (0.1, 0.01) in deme 2: stable dynamics occurs if migration *m *= 0.03.

When different demes have different values of one or more of the four parameters *b*, *c*, *u *and *s*, they oscillate at different frequencies (see (13) and Figures 1, 2). If the frequencies of the oscillations differ sufficiently between two or more of the populations and the demes are connected by migration (*i.e. *gene flow), the equilibrium points in all populations become stable rather than unstable (Figure 2b-d). Stabilisation of the oscillations is particularly responsive to differences in values of *b *and *u*, and to *s *when *s *is close to *u *(Figure 2b-d). Progress to stability is also determined by how far the initial gene frequencies are from the (unstable) equilibrium in each deme. In a system of two demes, if the initial frequencies are far from equilibrium in both demes, the dynamics are unstable in both locations (Figure 2e). If one or both demes are initially close to equilibrium, however, both of them can evolve to stable polymorphism if migration rates lie within an appropriate range (Figure 2f). Mathematically, this behaviour is explained by the existence of stable limit cycles [45]. This is biologically important because, when a new allele arises by mutation or by migration from a remote population, its frequency is almost invariably far from equilibrium. This does not destabilise the system, however, because all demes can evolve to a new stable equilibrium.

**Figure 3:**
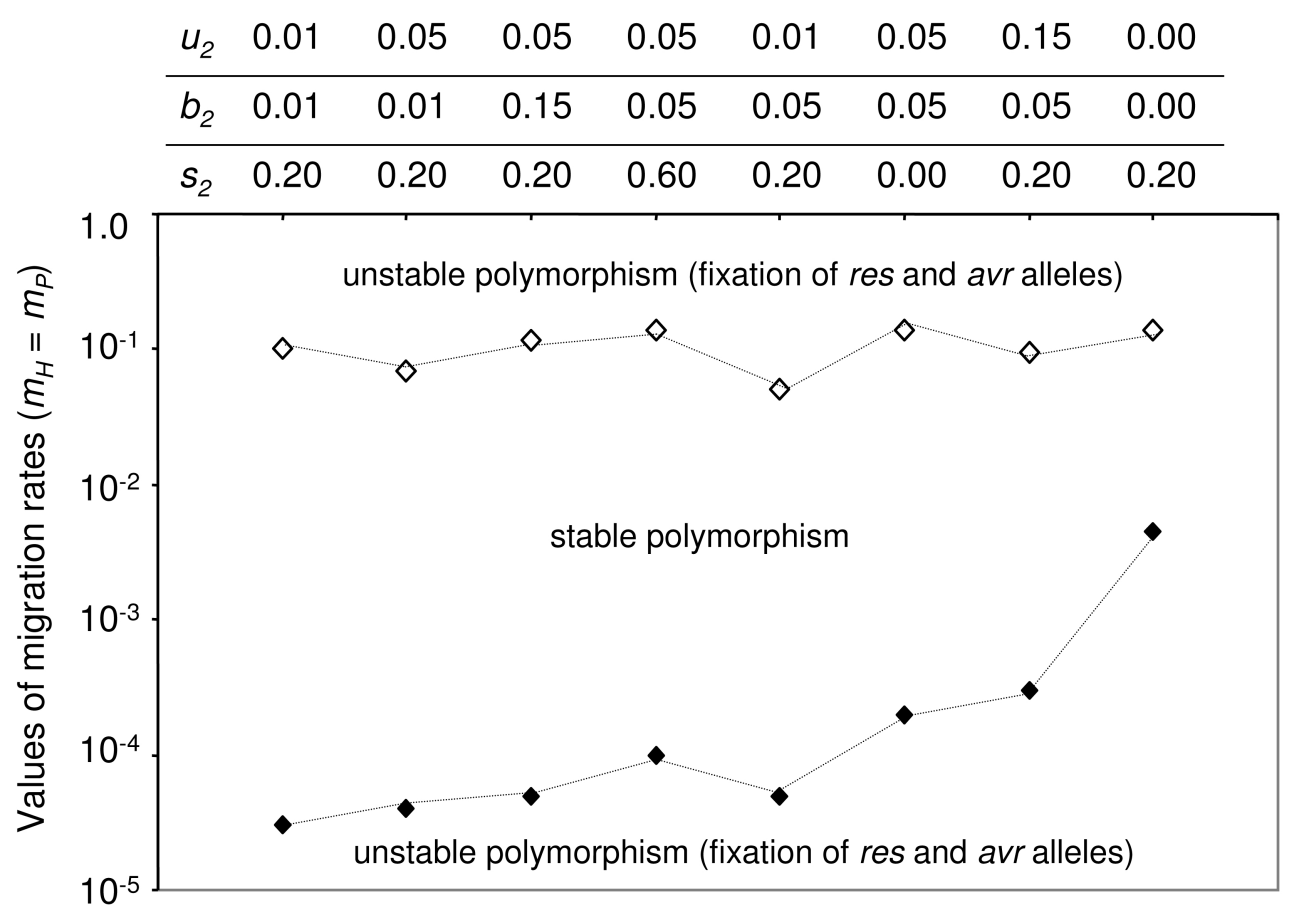
**Outcome of GFG coevolution in a two-deme model linked by migration, in relation to the values of parameters in deme 2 (top lines for *u_2_*, *b_2 _*and *s_2_*) and migration rates (host and parasite migration rates are equal, *m_P _*= *m_H_*)**. The behavior of the system was investigated for fixed parameters in deme 1: *u_1 _*= *b_1 _*= 0.05 and *s_1 _*= 0.2), and was consistent for three initial frequencies of the resistant and virulent alleles (*R_0 _*= *a_0_*): 0.05; 0.1 and 0.2. When the migration rate had values below the black diamond, or above the white diamond, the host susceptibility and parasite virulence alleles became fixed. At intermediate migration rates, stable polymorphism in host and parasite populations was observed (for simplicity, *c *= 1).

We investigated the occurrence of stable polymorphism quantitatively by simulating a model with fixed costs in deme 1 (*u_1 _*= *b_1 _*= 0.05 and *s_1 _*= 0.2). The costs of resistance and virulence were low, consistent with empirical evidence [46,47] and in line with previous theoretical studies [15,27]. The rates of migration (*m_P _*= *m_H_*) and costs in deme 2 (*u_2_*, *b_2 _*and *s_2_*) varied and the outcome of coevolution was recorded after 2,000 generations. The system was considered to be stable when the amplitude of the fluctuations of allele frequencies decreased over time and converged to an equilibrium value in both demes for three initial frequencies of resistant and virulent alleles (*R_0 _*= *a_0 _*= 0.05, 0.1 and 0.2 in both demes). This ensured that limit cycles occurred only for initial allele frequencies below 0.05. Combinations of parameter values in deme 2 were order in relation to the width of migration rate range, between the critical upper and lower values, which promoted stable polymorphism (Figure 3).

The stability (Figure 2b) or instability (Figure 2a) of the GFG system depended on the balance between the strength of selection and the migration rate (weak coupling, Figure 3). More precisely, stability arose with increasing difference between the natural periods of oscillation in the two populations (see (13, 14), Additional File 1, section 7). Asynchrony between oscillators is thus responsible for stability in GFG systems because it creates negative direct FDS (see (3) and Additional File 1, section7). In general, polymorphism occurred within an intermediate range of migration rates for given *b*, *u*, *s*. Below this range, demes behaved independently of one another and thus had unstable co-evolutionary dynamics (Figure 3). Above this range, all demes were synchronised to a frequency intermediate between the natural frequencies of each deme and the dynamics were unstable in all demes, with allele frequencies (*R*,*a*) spiralling outwards to fixation of virulent and susceptible alleles (Figure 2e, 3). Variation between demes for costs of virulence (*b_2 _*≠ *b_1 _*in Figure 3) was an important factor promoting stability. Variation between demes in the cost of being diseased (*s_2 _*≠ *s_1 _*in Figure 3) and cost of resistance (*u_2 _*≠ *u_1 _*in Figure 3) influenced the stability of polymorphism to a lesser extent. The smallest range of migration rates for which stable polymorphism was observed occurred for absence of costs in deme 2 (*u_2 _*= *b_2 _*= 0 in Figure 3). Finally, comparing *b_2 _*or *u_2 _*= 0.01, 0.05 and 0.15 in Figure 3, other things being equal, increasing costs of resistance or virulence in deme 2 above values in deme 1 did not necessarily favour stable polymorphism. This counter-intuitive result occurs because direct FDS is determined by the difference of the values of costs between demes in a non-linear manner (15-16).

**Figure 4:**
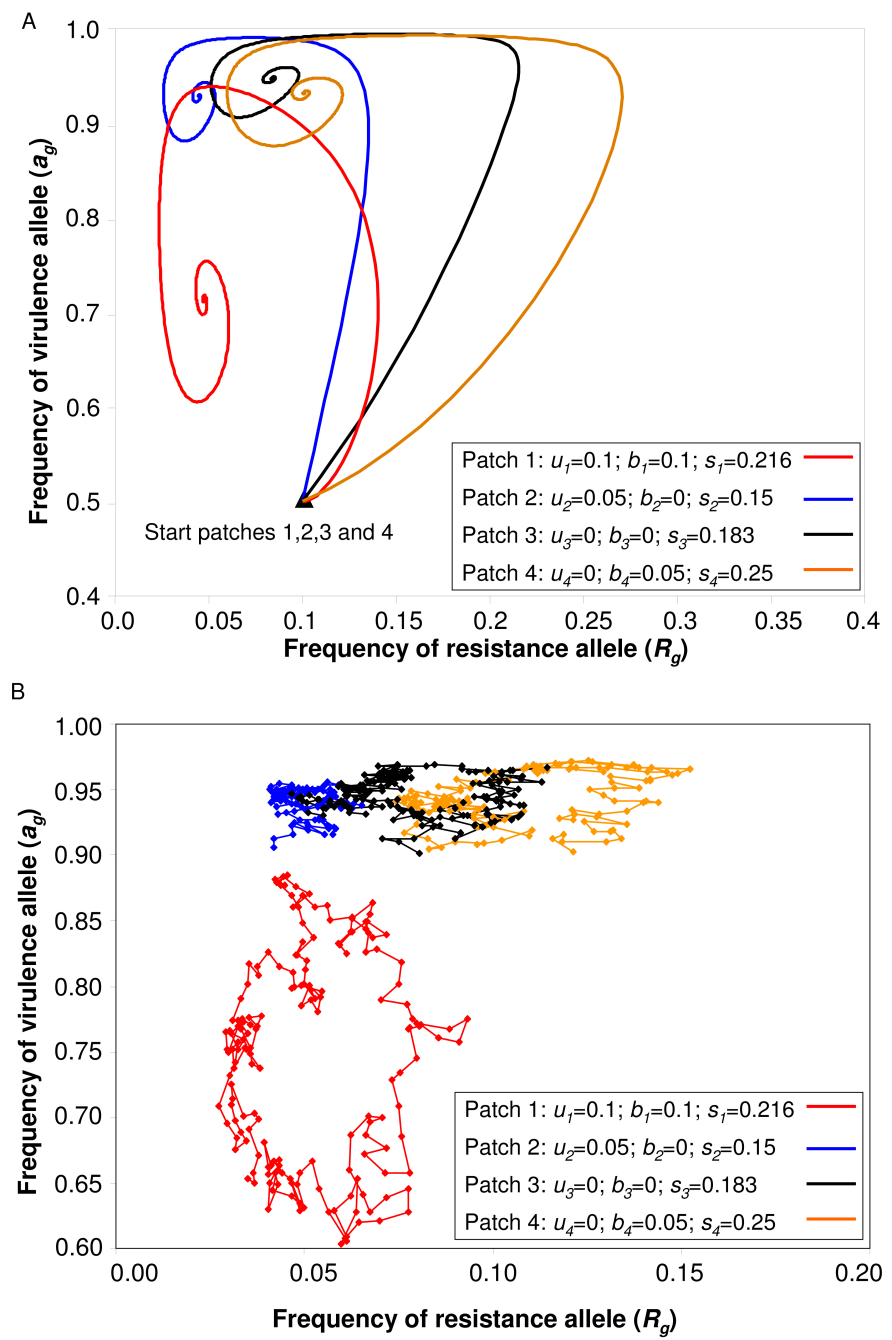
**Dynamics of allele frequencies in *n *= 4 patches with different costs of resistance and virulence**. Patches are characterised by variation of *b*, *u*, and *s*. Patch 1 (red) therefore represents a sub optimal environment for *RES *plants and *avr *parasites, while Patch 3 (black) represents an optimal environment for *RES *hosts and *avr *parasite as the costs of these alleles are zero. Patches 2 (blue) and 4 (orange) are respectively environment in which only *RES *and *avr *alleles are costly. In the four patches, the initial *RES *frequency is 0.1 and *avr *frequency is 0.5. The model was run for 1000 generations, and *m_H _*= *m_P _*= 0.05. a) Stable dynamics appear as inward spiralling towards the four different equilibrium points in an infinite deterministic population model. b) Trajectory of allele frequencies for one cycle in each patch, showing irregular, anti-clockwise cycling around the stable equilibrium in a stochastic finite population model (Only the last 200 generations over 1000 are shown).

The results of Figure 2, based on the system of equations (11, 12), can be generalised to a system with arbitrary number of demes, because stability occurs in all populations in a deterministic model (Figure 4). As shown analytically (8-9), the system can be stable both in all demes and in the whole metapopulation if resistance, virulence or both are cost-free in some (but not all) demes (Figure 3).

### Transient polymorphism in a homogeneous metapopulation with two demes

An interesting special case occurs in a metapopulation with a homogeneous environment when all demes have the same set of parameter values but the initial allele frequencies are distant from equilibrium and differ between demes. Examples are provided for this model based on the system of equations (8, 9) assuming *u_1 _*= *u_2_*, *b_1 _*= *b_2_*, and *s_1 _*= *s_2_*. There is first a transient phase in which allele frequencies move towards the unstable, interior equilibrium, driven by exchange of alleles between demes in which allele frequencies differ (Additional File 1, section 8). As frequencies converge on the equilibrium point, the dynamics in the two demes become synchronised, then the graph spirals outwards and alleles become fixed (Additional File 2, Figure S1). Increasing migration between demes shortens the length of the initial transient phase of quasi-stabilisation (Additional File 1, section 8).

### Simulation results for the GFG model with 4 demes

A stochastic version of the GFG model with four demes, based on equations (4, 5) illustrates a realistic situation with finite, variable host and parasite population sizes (here, both have size *N *= 1,000 individuals). At each host generation, a random number of individuals of each genotype is added to or removed from the populations, the maximum change being a fraction *F *(here, *F *= 0.05). For instance, the number of *RES *plants added in a given generation is: Δ*R = NRFσ *where *σ *is a random number from a uniform distribution between -1 and 1. The results (Figure 4b) are very similar to those of the deterministic model (Figure 4a). While allele frequencies spiral towards equilibrium, stochastic events nudge them away. This results in allele frequencies cycling around the theoretical equilibrium, particularly when genetic drift is limited. Higher values of genetic drift, and smaller population size (*N *smaller) lead to increased stochasticity and higher probability of allele fixation (not shown).

## Discussion

It has been proposed that spatial subdivision of populations maintains genetic diversity [32,37,38], in particular at genes controlling pathogenicity in parasites and resistance in hosts [30,39,40]. More specifically, it has been shown theoretically that spatial heterogeneity in a metapopulation can contribute to stabilising polymorphism in these genes if different demes have different environments, such that the coefficients of natural selection acting on the host or parasite vary between some or all demes [30,40]. The coefficients of natural selection include the cost to a host of being diseased [30,40] and, here, the genetic costs of host resistance and parasite virulence in a GFG system.

The theory reported here describes the genetic processes which underpin the stabilisation of polymorphism in coevolving hosts and parasites in a spatially structured population. It is shown that gene flow within a heterogeneous environment generates direct frequency-dependent selection, which can act together with the indirect frequency-dependent selection inherent in host-parasite interactions to generate long-term, balanced polymorphism at interacting host and parasite loci. This is consistent with the "trench warfare" scenario of host-parasite coevolution [2]. Note that the derivation of the mathematical conditions for the global stability of polymorphism and polymorphism in all demes lies beyond the present work, for which we refer readers to previous studies [34,37,38].

The fitness of host organisms depends on gene frequencies in their parasites and *vice-versa*. This process, which is well-understood [14,17], is an example of indirect FDS [15]. Together with costs of resistance (*u*) and virulence (*b*), it causes the frequencies of interacting host and parasite alleles to cycle. The oscillations are centred on an equilibrium point at which there is polymorphism at the corresponding loci in the two species (Figure 2a).

### Direct Frequency-dependent selection

Gene flow in a heterogeneous environment generates direct frequency-dependent selection, such that the selective advantage of an allele in either species declines as that allele becomes more common (3). This has been described as balanced polymorphism due to heterogeneous habitats or migration-selection balance [34,38,48]. In the models of host-parasite coevolution analysed here, direct FDS acts together with indirect FDS to cause the cycles of gene frequencies to stabilise at a state of balanced polymorphism (Figures 2b-d, 2f). In the absence of ecological or epidemiological processes which generate direct FDS, the equilibrium is unstable and the oscillations expand outwards resulting ultimately in fixation of alleles in both species (Figures 2a, e). When demes have different environmental conditions, resulting in different values of *u*, *b *or the cost to a plant of being diseased (*s*), the periods of the cycles of gene frequencies differ (13). In accordance with the general results from [32,34,37,48] in single species models and those of Nuismer for a GFG model [40], we show that greater difference in these coevolutionary parameters between two or more demes generates more strongly negative direct FDS and therefore a greater potential for stability of the polymorphic equilibrium point. Our first important result (3) therefore demonstrates that migration-selection balance is a case of direct FDS. Note, however, that each deme's equilibrium point is only stable over a range of low migration rates [34,37,48]. This principle also applies widely to biological interactions, since prey and predator numbers can also be stabilised by damping of oscillations in their numbers, for example if there is spatial variation such as a gradient of birth rates [49].

Damgaard [18] investigated a GFG model in a metapopulation where stable polymorphism occurs without costs of virulence or resistance (*u *= *b *= 0 in all demes). That model has spatially heterogeneous incidence of a second parasite species in a metapopulation with high rates of extinction and recolonisation of demes, and recolonisation of patches *via *a seed bank. We suggest that this model favours the occurrence of stable polymorphism because it comprises two features generating direct FDS, disease severity varying in space due to the prevalence of a second parasite (a specific example of spatial variation in costs relevant to the present paper) and a seed bank [27].

### Role of costs of resistance and virulence

Costs of resistance (*u*) and virulence (*b*) are required to drive the cyclical dynamics of host and parasite gene frequencies in the GFG model. A positive value of *u *reduces the frequency of resistance when virulence is common (a>â) while a positive value of *b *reduces the frequency of virulence when resistance is rare ($R<\hat{R}$). Our second important result states that, in a metapopulation, direct FDS is generated such that polymorphism can be stable even if one or both of these costs is zero in some demes, so long as they each have positive values in at least one deme (Figures 3, 4b). This implies that the observation of polymorphism in a given deme does not imply that the *RES *and *avr *alleles are costly in that deme. Instead, the deme may be linked by migration to other, possibly unobserved demes where *b*, *u *or both are positive (8-9). This is consistent with the lack of empirical evidence for high costs of *RES *and *avr *alleles [22,46], with a few significant exceptions [21,25,50].

A third important result is that heterogeneity in costs of virulence (*b*) and to a lesser extent resistance (*u*) alleles are the main drivers for generating direct FDS and stability of polymorphism (Figure 3). In fact, variability among demes only for the cost for a plant of being diseased (*s *in Figure 3) may not create strong differences in oscillations between demes (14). Models [30,39,40] and empirical tests [5-7,41,51] of the geographic mosaic of coevolution have highlighted the importance of variation for disease severity or presence/absence of parasites for driving the coevolutionary dynamics (hot and cold spots of coevolution). Most observations of the coevolutionary dynamics have thus focused on revealing heterogeneity for disease severity or disease presence depending on local ecological conditions (*e.g. *[52]) and genotype-by-genotype-by-environment interactions [53]. Our results indicate, however, that to understand the mechanisms maintaining genetic variability at plant resistance and parasite virulence genes it would also be fruitful to study variation in the costs of virulence [23,24] or resistance [22] alleles in different environments. Recent experimental evidence for variability of costs has paved the way for future empirical tests of similar variability in natural populations. However, little is known as yet about the genetic mechanisms generating variation in costs in response to physical characteristics of habitats, such as temperature or humidity. Our results also suggest that variability in levels of partial resistance between demes (*c *< 1 in (4, 5) and Table 1) may not create strong differences in oscillations between demes (13), and thus may not be a main determinant in promoting stable polymorphism in GFG systems [44].

### Metapopulation with homogeneous environment

In a metapopulation in which all demes have identical environments, transient polymorphism can be generated in two ways. Firstly, as shown here, unstable dynamics can create an impression of stable polymorphism across the metapopulation (Additional File 2, Figure S1; [30]); this has been described as 'statistical polymorphism' in prey-predator models [54]. In a metapopulation with a high rate of population extinction, recolonisation of demes produces large, random variation in allele frequencies between populations, sustaining transient polymorphism and thus increasing the lifetime of alleles [20,30], especially when there are several interacting pairs of *RES *and *AVR *genes [19,20,55]. Asynchrony can arise between identical coupled oscillators, providing that each one has a specific noise function [56]. This implies that negative direct FDS may arise in a homogeneous metapopulation if each deme exhibits high levels of random processes such as drift and extinction-recolonisation with different statistical characteristics [20,30]. Note that the model of Frank [55] maintains polymorphism in an homogeneous metapopulation by considering density-dependent disease transmission following a Lotka-Volterra model for multiple GFG loci in host and parasite. Density-dependence disease transmission, central to ecological feedback models [57], generates negative direct FDS and thus to stabilises GFG polymorphism in single populations [27].

Secondly, when host and parasite migration is limited to adjacent demes, and initial frequencies differ between populations, waves of genotypes can spread in the metapopulation [19,30]. Asynchrony can then be maintained by a few patches which are out of phase with the rest of the metapopulation and act as pacemakers [19,30], potentially leading to damping of local oscillations and stabilisation of gene frequencies, analogous to the process shown in Figure 4. Polymorphism is thus present in the metapopulation as a whole, but not in each deme. It remains for empirical studies to be designed to test the existence of such phenomena (but see [10]).

### Observing GFG coevolution

Observation of the long-term dynamics of polymorphism at interacting host and parasite genes and empirical tests to distinguish the "trench warfare" and "arms race" scenarios require either the collection of very long time series for host and parasite populations [58] or the inference of past evolutionary events from sequence data [2]. The increasing availability of genomic data for multiple genes and multiple populations may allow tests of a wide range of complex coevolutionary scenarios (*e.g. *[59]) arising from heterogeneous abiotic conditions in space and genotype-by-genotype-by-environment interactions [53].

The most promising body of empirical research testing coevolutionary scenarios analogous to our theory comes from controlled laboratory experiments of coevolving populations of phages (*e.g. *Φ2, PP7, T7) and bacteria (e.g. *Pseudomonas fluorescens, P. aeruginosa, Escherichia coli*). Even though bacteria-phage systems may not present GFG interactions, but may have inverse-GFG relationships [60], coevolutionary dynamics are observed in those experiments. The influence of key components of the theory on geographic mosaic of coevolution on actual coevolutionary dynamics has been empirically tested. These factors include for example gene flow across a spatially structured landscape with productivity gradients [5-8], the importance of migration of hosts from parasite-free demes [41], or the effect of dispersal from hot-spots to cold spots and *vice versa *on the speed of coevolution [10].

These experimental designs of structured heterogeneous populations with gene flow can be adapted to test experimentally key predictions from GFG models. Genetic variation in the costs of bacterial resistance or viral virulence alleles can be quantified (as has been done for example with pepper-infecting tobamoviruses, [25]) across environments (see [9,61,62]). Values of host fitness reduction upon infection determine both the expected frequencies of coevolutionary cycles (equation 13) and the occurrence of stable polymorphism in different demes linked by migration [42,62]. This study indicates that heterogeneous costs of resistance or virulence between demes (even zero costs in some but not all demes) is a more important determinant of stability in a coevolving host-parasite interaction rather than heterogeneous distribution of the cost of being diseased. Our results imply that in a homogeneous metapopulation, high gene flow synchronises all demes to the most unstable dynamics, a phenomenon which can be tested empirically (for example see [10,51]). Finally, our simulations suggest that in an heterogeneous metapopulation, each deme presents a specific coevolutionary dynamics characterized by its equilibrium point and frequency of oscillations (Figure 4). Our results agree thus with theoretical [30,58] and empirical [62] studies implying that host-parasite adaptation may not be positively correlated in space and time.

Finally, our GFG model assumes that epidemiological processes are density-independent, and makes the simplifying assumption that population sizes are equal between demes. When realistic host-parasite systems are considered, one may expect parasite and host dispersal to depend on the density of susceptible and infected hosts in each patch. Fluctuations in host density across patches would thus potentially affect patterns of local adaptation [20,29,40] and maintenance of stable polymporphism. Future analysis of coevolution would thus benefit from integrating epidemiological processes and ecological feedback (e.g. [57]) into current GFG models.

## Conclusions

The general principle that migration in heterogeneous metapopulations can stabilise polymorphism in GFG interactions is applicable to any system of victims and exploiters, including interactions between animals and parasites, predators and prey, and hosts and parasitoids [30,48,49]. It provides a mechanism to drive the maintenance of biological diversity in models of the geographic mosaic of co-evolution [39]. We predict that the GFG polymorphisms which are observable in nature may involve genes which have fitness costs that vary due to varying abiotic or biotic conditions in space (between demes, [22-24]) or in time (temporal change, [63]). Our results also support the view that monoculture in arable or livestock farming increases the risk and potential severity of disease [64], not only because genetic diversity is limited but also because agricultural environments are simplified and generally uniform.

## Authors' contributions

AT and JKMB designed and performed research, and wrote the paper. All authors read and approved the final manuscript.

## Supplementary Material

Additional file 1 **Additional Information for analytical results**. The file contains 8 sections describing details of the analytical derivations. Click here for file

Additional file 2 **Figure S1**. The file contains the Figure S1. Click here for file
