# Supplementary material for: Spatial heterogeneity, frequency-dependent selection and polymorphism in host-parasite interactions
Source: BMC Evol Biol. 2011 Nov 1;11:319. doi: 10.1186/1471-2148-11-319 (PMC3273489; doi:10.1186/1471-2148-11-319)
Supplement: Additional file 1 — Additional Information for analytical results. The file contains 8 sections describing details of the analytical derivations. [file 1471-2148-11-319-S1.PDF]

Spatial heterogeneity and polymorphism in host-parasite interactions

Aurélien Tellier [1] and James K.M. Brown [2]

[1] *Section of Evolutionary Biology, Biocenter, University of Munich, 82152 Planegg-Martinsried, Germany*

[2] *Department of Disease and Stress Biology, John Innes Centre, Colney, Norwich, NR4 7UH, UK*

Additional File 1

Section 1. Direct frequency-dependent selection (on any gene) generated by migration between two demes with different environments is negatively correlated with allele frequency.

Section 2. Direct frequency-dependent selection is also generated by migration between demes with different environments if migration takes place before selection.

Section 3. Dependence of parasite equilibrium allele frequencies on host migration and *vice-versa*

Section 4. Direct frequency-dependent selection on resistance and avirulence genes generated by migration between demes with different environments.

Section 5: Conditions for stability of the interior equilibrium point

Section 6: Frequency of oscillations of the simple gene-for-gene system close to the unstable interior, non-trivial equilibrium.

Section 7: The strength of direct frequency-dependent selection depends on the difference of oscillation periods in a two-deme meta-population

Section 8: The special case of a homogeneous environment

Section 1: Direct frequency-dependent selection (on any gene) generated by migration between two demes with different environments is negatively correlated with allele frequency.

We assume that migration takes place at the end of each generation, which is typical of many organisms; for example, seeds of higher plants and sexual spores of fungi are dispersed at the end of the annual life cycle. Selection within each deme therefore precedes migration between demes. We consider a locus with two alleles, *G* and *g*, with frequencies G_i and g_i in deme *i*. For the sake of generality and for applicability to models of coevolution, we consider natural selection acting on both alleles. After selection, the allele frequencies are:

$$\tilde{G}_i = \frac{G_i(1-\tau_i)}{1-G_i\tau_i - g_i\sigma_i} \text{ and } \tilde{g}_i = \frac{g_i(1-\sigma_i)}{1-G_i\tau_i - g_i\sigma_i} \qquad \text{S1.1}$$

At the end of a generation, a proportion m_{ji} of the population in deme *i* is formed by migrants from deme *j*, while a proportion L_i originate in deme *i* itself, i.e. $L_i = m_{ii} = 1-\sum_j m_{ji}$ (all populations are assumed to be large enough for random genetic drift to be entirely negligible). Migration takes place after selection. The recurrence equation for the ratio of G_i to g_i between generations is:

$$\frac{G_i'}{g_i'} = \frac{\tilde{G}_i L_i + \sum_{j\neq i} \tilde{G}_j m_{ji}}{\tilde{g}_i L_i + \sum_{j\neq i} \tilde{g}_j m_{ji}} = \frac{\dfrac{G_i(1-\tau_i)L_i}{1-G_i\tau_i - g_i\sigma_i} + \sum_{j\neq i}\dfrac{G_j(1-\tau_j)m_{ji}}{1-G_j\tau_j - g_j\sigma_j}}{\dfrac{g_i(1-\sigma_i)L_i}{1-G_i\tau_i - g_i\sigma_i} + \sum_{j\neq i}\dfrac{g_j(1-\sigma_j)m_{ji}}{1-G_j\tau_j - g_j\sigma_j}} \qquad \text{S1.2}$$

For given G_j ($j\neq i$), S1.2 can be rewritten as

$$\left(\frac{G_i}{g_i}\right)' = \frac{G_i(1-\tau_i)L_i + \phi_i(1-G_i\tau_i - g_i\sigma_i)}{g_i(1-\sigma_i)L_i + \psi_i(1-G_i\tau_i - g_i\sigma_i)} \qquad \text{S1.3}$$

where

$$\phi_i = \sum_{j\neq i}\frac{G_j(1-\tau_j)m_{ji}}{1-G_j\tau_j - g_j\sigma_j} \text{ and } \psi_i = \sum_{j\neq i}\frac{g_j(1-\sigma_j)m_{ji}}{1-G_j\tau_j - g_j\sigma_j} \qquad \text{S1.4}$$

Transforming allele frequencies to the logit of one of the alleles, i.e. γ_i=log(G_i/g_i), has two advantages. Firstly, the rate of change of γ_i over time is constant when the rate of natural selection is constant and, in particular, independent of allele frequencies. Secondly, it simplifies the algebra considerably. The change between generations is therefore (noting that $G_i + g_i = 1$ to simplify the algebra):

$$\Delta\gamma_i = \gamma_i' - \gamma_i = \log\left(\frac{G_i'}{g_i'}\right) - \log\left(\frac{G_i}{g_i}\right)$$

$$\Delta\gamma_i = \log\left\{\frac{G_i(1-\tau_i)L_i + \phi_i(1-G_i\tau_i - g_i\sigma_i)}{g_i(1-\sigma_i)L_i + \psi_i(1-G_i\tau_i - g_i\sigma_i)}\right\} - \gamma_i$$

$$= \log\left\{\frac{(1-\tau_i)L_i + \phi_i\left[\sigma_i - \tau_i + G_i^{-1}(1-\sigma_i)\right]}{(1-\sigma_i)L_i + \psi_i\left[\tau_i - \sigma_i + g_i^{-1}(1-\tau_i)\right]}\right\} \qquad \text{S1.5}$$

The sign of the differential $d\Delta\gamma_i/d\gamma_i$ describes the direction of direct frequency-dependent selection (dFDS) in deme i, with given allele frequencies in the other demes. If the intensity of natural selection is independent of the allele frequencies in deme i, G_i and g_i, $d\Delta\gamma_i/d\gamma_i=0$. By the chain rule,

$$\frac{d\Delta\gamma_i}{d\gamma_i} = \frac{d_i\Delta\gamma_i}{dG_i}\cdot\frac{dG_i}{d\gamma_i} \qquad \text{S1.6}$$

Note that $dG_i/d\gamma_i = G_i g_i$ because

$$\gamma_i = \frac{G_i}{1-G_i} \Leftrightarrow G_i = \frac{e^{\gamma_i}}{1+e^{\gamma_i}} \Rightarrow \frac{dG_i}{d\gamma_i} = \frac{e^{\gamma_i}}{\left(1+e^{\gamma_i}\right)^2} = G_i g_i \qquad \text{S1.7}$$

It can be seen by inspection of the second line of S1.5 that $d\Delta\gamma_i/d\gamma_i$ is negative because $\Delta\gamma_i$ declines with increasing G_i. To be precise,

$$\frac{d\Delta\gamma_i}{d\gamma_i} = -\left(\begin{array}{l}\dfrac{g_i\phi_i(1-\sigma_i)}{G_i\left\{(1-\tau_i)L_i + \phi_i\left[\sigma_i - \tau_i + G_i^{-1}(1-\sigma_i)\right]\right\}} \\ +\dfrac{G_i\psi_i(1-\tau_i)}{g_i\left\{(1-\sigma_i)L_i + \psi_i\left[\tau_i - \sigma_i + g_i^{-1}(1-\tau_i)\right]\right\}}\end{array}\right) \qquad \text{S1.8}$$

When there is no migration into deme i from any other deme, all $m_{ji} = 0 \Rightarrow m_{ii} = L_i = 1$. From S1.4 this implies, $\phi_i=\psi_i=0$, so $d\Delta\gamma_i/d\gamma_i=0$ when $L_i = 1$. Also, when the coefficients of natural selection, σ and τ, are both constant across demes and the frequencies of alleles G and g are the same in all demes, $d\Delta\gamma_i/d\gamma_i=0$; this is most easily seen in S1.2.

$d\Delta\gamma_i/d\gamma_i$ cannot be positive unless ϕ_I and ψ_i are very large and σ_i and τ_i are very different, while it is only zero under the particular circumstances described above. In practical situations, therefore, there is always negative dFDS in deme i, with the net intensity of natural selection for an allele declining as that allele becomes more common. The only conditions for this to occur are that there must be migration from other demes into deme i and that the fitness value of alleles must differ between two or more populations.

Stable polymorphism can result, with both G and g alleles maintained in the population, if at some value of G_i: $0<G_i<1$, the fitness values of the two alleles are equal, so that $\Delta\gamma_i=0$, with $\Delta\gamma_i>0$ below that value of G_i and $\Delta\gamma_i<0$ above that value.

These results apply to any gene, not just those involved in host-parasite or other victim-exploiter interactions. The exact conditions for maintenance of polymorphism in a two patch model were given by (Prout 1968; Maynard-Smith 1970; Bulmer 1972; Strobeck 1974; Gliddon & Strobeck 1975; Karlin & Campbell 1980; Nagylaki & Lou 2006; Nagylaki & Lou 2007; Star et al. 2007a; Star et al. 2007b).

Section 2: Direct frequency-dependent selection is also generated by migration between demes with different environments if migration takes place before selection.

If migration takes place before selection, the allele frequencies after migration are:

$$\hat{G}_i = G_i L_i + \sum G_j m_i \text{ and } \hat{g}_i = \sum g_j m_j \qquad \text{S2.1}$$

Let $\tilde{G}_i$ then be the frequency of G_i in deme i after selection has taken place following migration.

$$\frac{G_i'}{g_i'} = \frac{\tilde{G}_i L_i + \sum_{j \neq i} \tilde{G}_j m_{ji}}{\tilde{g}_i L_i + \sum_{j \neq i} \tilde{g}_j m_{ji}} = \frac{\left(G_i L_i + \sum_{j \neq i} G_j m_{ji} \right)(1-\tau_i)}{\left(g_i L_i + \sum_{j \neq i} g_j m_{ji} \right)(1-\sigma_i)} \qquad \text{S2.2}$$

The change in γ_i, the logit of G_i, is:

$$\begin{aligned} \Delta\gamma_i &= \log\left\{ \frac{(G_i L_i + \zeta_i)(1-\tau_i)}{(g_i L_i + \xi_i)(1-\sigma_i)} \right\} - \gamma_i \\ &= \log\left\{ \frac{(L_i + \zeta_i G_i^{-1})(1-\tau_i)}{(L_i + \xi_i g_i^{-1})(1-\sigma_i)} \right\} \end{aligned} \qquad \text{S2.3}$$

where

$$\zeta_i = \sum_{j \neq i} G_j m_{ji} \text{ and } \xi_i = \sum_{j \neq i} g_j m_{ji}$$

The differential of this expression with respect to γ_i is negative:

$$\begin{aligned} \frac{d\Delta\gamma_i}{d\gamma_i} &= \frac{d}{d\gamma_i}\left[\log\left(L_i + \zeta_i G_i^{-1}\right) - \log\left(L_i + \xi_i g_i^{-1}\right) \right] \\ &= G_i g_i \left[-\frac{\zeta_i G_i^{-2}}{L_i + \zeta_i G_i^{-1}} - \frac{\xi_i g_i^{-2}}{L_i + \xi_i g_i^{-1}} \right] \\ &= -\frac{\zeta_i g_i}{L_i G_i + \zeta_i} - \frac{\xi_i G_i}{L_i g_i + \xi_i} \end{aligned} \qquad \text{S2.4}$$

Hence migration between demes with different environments, followed by selection within the target deme, always generates negative direct frequency-dependent selection.

Note that the frequency-dependence term does not depend on the selection coefficients within the target deme. This can be understood intuitively because the contribution of genes from demes $j \neq i$, which 'dilutes' selection for allele G in deme i depends only on migration rates, not on variation between selection coefficients among demes.
Similar results are shown for example in (Nagylaki 1992).

Section 3: Dependence of parasite equilibrium allele frequencies on host migration and *vice-versa*

Resistance and virulence frequencies at equilibrium in a simple GFG model for a single population are found given by Eq. 5 in the text. In this section, we calculate values of the allele frequencies at equilibrium if one of the two species migrates between demes but the other does not.

The recurrence equations for R and A in one population (eq. 4 in paper) can both be written in the form

$$\frac{X'}{x'} = \frac{X(\varphi + \chi Y)}{x\psi} \quad \text{S3.1}$$

where X and Y are frequencies of R and A or *vice-versa*, x=1–X and φ, χ and ψ are constants. At equilibrium in one population i,

$$\frac{\hat{R}_i}{\hat{r}_i} = \frac{\hat{R}_i(1-u_i)(1-s_i+s_i\hat{A}_i)}{\hat{r}_i(1-s_i)} = \frac{\hat{R}_i(\varphi_i+\chi_i\hat{A}_i)}{\hat{r}_i\psi_i} \quad \text{S3.2}$$

where

$$\left.\begin{aligned} \varphi_i &= (1-u_i)(1-s_i) \\ \chi_i &= (1-u_i)s_i \\ \psi_i &= 1-s_i \end{aligned}\right\} \quad \text{S3.3}$$

Therefore,

$$\hat{A}_i = \frac{u_i(1-s_i)}{(1-u_i)s_i} = \frac{\psi_i - \varphi_i}{\chi_i} \quad \text{S3.4}$$

We consider two connected populations of equal size at equilibrium with rates of host and parasite migration m_H and m_p respectively. We also assume that selection and migration precede reproduction. Therefore,

$$\frac{\tilde{R}_i}{\tilde{r}_i} = \frac{(1-m_H)\tilde{R}_i(\varphi_i+\chi_i\tilde{A}_i)+m_H\tilde{R}_j(\varphi_j+\chi_j\tilde{A}_j)}{(1-m_H)\tilde{r}_i\psi_i+m_H\tilde{r}_j\psi_j} \quad \text{S3.5}$$

($\tilde{R}$ notation as in SI1). If there is no migration of the host ($m_H = 0$),

$$\frac{\tilde{R}_i}{\tilde{r}_i} = \frac{\tilde{R}_i(\varphi_i+\chi_i\tilde{A}_i)}{\tilde{r}_i\psi_i} \Rightarrow \tilde{A}_i = \frac{\psi_i-\varphi_i}{\chi_i} = \hat{A}_i \quad \text{S3.6}$$

Hence the deviation of the equilibrium value of A ($\tilde{A}$) in each of two populations from that in a single unconnected population ($\hat{A}$) depends on migration of the host population if the

parasite does not migrate. By symmetry, the similar deviation of *R* depends on parasite migration if the host does not migrate.

Developing this analysis, the equilibrium values of *A* and *R* can be found when there is no migration of the parasite or the host, respectively. First, assume m_p=0 so that $\tilde{R}_i = \hat{R}_i$ in each deme *i*. Solving S3.5 for $\hat{A}_i$,

$$\tilde{A}_i = \hat{A}_i + \frac{m_H}{\chi_i(1-m_H)}\left\{\frac{\hat{r}_j}{\hat{r}_i}\psi_j - \frac{\hat{R}_j}{\hat{R}_i}\left(\varphi_j + \chi_j\tilde{A}_j\right)\right\} \quad \text{S3.7}$$

and by symmetry

$$\tilde{A}_j = \hat{A}_j + \frac{m_H}{\chi_j(1-m_H)}\left\{\frac{\hat{r}_i}{\hat{r}_j}\psi_i - \frac{\hat{R}_i}{\hat{R}_j}\left(\varphi_i + \chi_i\tilde{A}_i\right)\right\} \quad \text{S3.8}$$

Substituting S3.8 into S3.7,

$$\begin{aligned}&\tilde{A}_i\left(1 - \frac{m_H}{\chi_i(1-m_H)}\frac{\hat{R}_j}{\hat{R}_i}\chi_j\frac{m_H}{\chi_j(1-m_H)}\frac{\hat{R}_i}{\hat{R}_j}\chi_i\right) = \\ &\hat{A}_i + \frac{m_H}{\chi_i(1-m_H)}\left(\frac{\hat{r}_j}{\hat{r}_i}\psi_j - \frac{\hat{R}_j}{\hat{R}_i}\left[\varphi_j + \chi_j\left\{\hat{A}_j + \frac{m_H}{\chi_j(1-m_H)}\left(\frac{\hat{r}_i}{\hat{r}_j}\psi_i - \frac{\hat{R}_i}{\hat{R}_j}\varphi_i\right)\right\}\right]\right)\end{aligned} \quad \text{S3.9}$$

Simplifying S3.9, we eventually find

$$\tilde{A}_i = \hat{A}_i + \frac{m_H}{\chi_i(1-2m_H)}\left\{\psi_j(1-m_H)\left(\frac{\hat{r}_j}{\hat{r}_i} - \frac{\hat{R}_j}{\hat{R}_i}\right) + \psi_i m_H\left(1 - \frac{\hat{R}_j}{\hat{R}_i}\frac{\hat{r}_i}{\hat{r}_j}\right)\right\} \quad \text{S3.10}$$

Substituting S3.3 and S3.4 into S3.10,

$$\tilde{A}_i = \hat{A}_i + \frac{m_H}{(1-u_i)s_i(1-2m_H)}\left\{(1-s_j)(1-m_H)\left(\frac{\hat{r}_j}{\hat{r}_i} - \frac{\hat{R}_j}{\hat{R}_i}\right) + (1-s_i)m_H\left(1 - \frac{\hat{R}_j}{\hat{R}_i}\frac{\hat{r}_i}{\hat{r}_j}\right)\right\} \quad \text{S3.11}$$

An identical approach leads to a solution of the host equilibrium gene frequencies when there is migration of the parasite but not the host:

$$\tilde{R}_i = \hat{R}_i - \frac{m_p}{c_i(1-2m_p)}\left\{(1-b_j)(1-m_p)\left(\frac{\hat{a}_j}{\hat{a}_i} - \frac{\hat{A}_j}{\hat{A}_i}\right) + (1-b_i)m_p\left(1 - \frac{\hat{A}_j}{\hat{A}_i}\frac{\hat{a}_i}{\hat{a}_j}\right)\right\} \quad \text{S3.12}$$

Section 4: Direct frequency-dependent selection on resistance and avirulence genes generated by migration between demes with different environments.

Extending the notation of the Supporting Information, Section 1 (SI1), subscripts *H* and *P* for the the host and parasite *L* and *m* refer to the migration parameters respectively. Note that R_i is the frequency of resistant alleles at generation *t*, and R_i' at generation $t+1$. The recurrence equation for the ratio of resistant (*RES*) to susceptible (*res*) hosts is:

$$\frac{R_i'}{r_i'} = \frac{R_i(1-u_i)(1-s_i a_i)L_{Hi} + \sum_{j\neq i} R_j(1-u_j)(1-s_j a_j)m_{Hji}}{r_i(1-s_i)L_{Hi} + \sum_{j\neq i} r_j(1-s_j)m_{Hji}} \qquad \text{S4.1}$$

Using the notation of SI1, $\tilde{R}_i = R_i(1-u_i)(1-s_i a_i)$ and $\tilde{r}_i = r_i(1-s_i)$, the change in $\rho_i=\log(R_i/r_i)$ is

$$\Delta\rho_i = \log\left(\tilde{R}_i L_{Hi} + \sum_{j\neq i}\tilde{R}_j m_{Hji}\right) - \log\left(\tilde{r}_i L_{Hi} + \sum_{j\neq i}\tilde{r}_j m_{Hji}\right) - \rho_i \qquad \text{S4.2}$$

If there is no migration ($m_{Hji}=0$, $L_{Hi}=1$) or if the frequencies of *R* and *a* and the values of *u* and *s* are the same in all populations, S4.2 simplifies to $\Delta\rho_i = \tilde{\rho}_i - \rho_i$, so $d\Delta\rho_i / d\rho_i = 0$ and there is no dFDS on the *RES* locus. In other circumstances, by the chain rule,

$$\frac{d\Delta\rho_i}{d\rho_i} = \frac{d_i\Delta\rho_i}{dR_i}\cdot\frac{dR_i}{d\rho_i} \qquad \text{S4.3}$$

As $dR_i / d\rho_i = R_i r_i$,

$$\frac{d\Delta\rho_i}{d\rho_i} = R_i r_i \left\{ \frac{(1-u_i)(1-s_i a_i)L_{Hi}}{\tilde{R}_i L_{Hi} + \sum_{j\neq i}\tilde{R}_j m_{Hji}} + \frac{(1-s_i)L_{Hi}}{\tilde{r}_i L_{Hi} + \sum_{j\neq i}\tilde{r}_j m_{Hji}} \right\} - 1 \qquad \text{S4.4}$$

Simplification of S4.4 leads to

$$\frac{d\Delta\rho_i}{d\rho_i} = -\frac{\tilde{R}_i R_i L_{Hi}\sum_{j\neq i}\tilde{r}_j m_{Hji} + \tilde{r}_i r_i L_{Hi}\sum_{j\neq i}\tilde{R}_j m_{Hji} + \left(\sum_{j\neq i}\tilde{R}_j m_{Hji}\right)\left(\sum_{j\neq i}\tilde{r}_j m_{Hji}\right)}{\left(\tilde{R}_i L_{Hi} + \sum_{j\neq i}\tilde{R}_j m_{Hji}\right)\left(\tilde{r}_i L_{Hi} + \sum_{j\neq i}\tilde{r}_j m_{Hji}\right)} \qquad \text{S4.5}$$

Like S1.8, this differential is never positive. It is negative at equilibrium, implying that there is negative direct FDS on the *RES* gene if there is migration between population *i* and other populations and if the fitness cost of resistance (*u*) or the cost of disease (*s*) differs between at least some populations.

This can also be interpreted biologically as follows for a simple two-deme model. Suppose that in deme 1, values of the parasite fitness parameters *b* and *c* are such that there is a higher frequency of the *RES* allele than in deme 2. Increasing selection for *RES* allele leads to a higher proportionate rate of migration of *res* alleles from deme 2 into deme 1, and *vice-versa* for deme 2. This causes a net decrease in selection for *RES* in deme 1 and for *res* in deme 2.

Similarly, considering the parasite, the recurrence equation for the ratio of *AVR* to *avr* frequencies is:

$$\frac{A_i'}{a_i'}=\frac{A_i\left(1-c_iR_i\right)L_{Pi}+\sum_{j\neq i}A_j\left(1-c_jR_j\right)m_{Pji}}{a_i\left(1-b_i\right)L_{Pi}+\sum_{j\neq i}a_j\left(1-b_j\right)m_{Pji}} \qquad \text{S4.6}$$

Writing $\tilde{A}_i=A_i\left(1-c_iR_i\right)$ and $\tilde{a}_i=a_i\left(1-b_i\right)$, the change in α_i=log(A_i/a_i) is

$$\Delta\alpha_i=\log\left(\tilde{A}_iL_{Pi}+\sum_{j\neq i}\tilde{A}_jm_{Pji}\right)-\log\left(\tilde{a}_iL_{Pi}+\sum_{j\neq i}\tilde{a}_jm_{Pji}\right)-\alpha_i \qquad \text{S4.7}$$

Under similar conditions to those for equation S4.3, the equation that describes direct FDS on the *AVR* locus is

$$\begin{aligned}\frac{d\Delta\alpha_i}{d\alpha_i}&=A_ia_i\left\{\frac{\left(1-c_iR_i\right)L_{Pi}}{\tilde{A}_iL_{Pi}+\sum_{j\neq i}\tilde{A}_jm_{Pji}}+\frac{\left(1-b_i\right)L_{Pi}}{\tilde{a}_iL_{Pi}+\sum_{j\neq i}\tilde{a}_jm_{Pji}}\right\}-1\\&=\frac{-\tilde{A}_iA_iL_{Pi}\sum_{j\neq i}\tilde{a}_jm_{Pji}-\tilde{a}_ia_iL_{Pi}\sum_{j\neq i}\tilde{A}_jm_{Pji}-\sum_{j\neq i}\tilde{A}_jm_{Pji}\sum_{j\neq i}\tilde{a}_jm_{Pji}}{\left(\tilde{A}_iL_{Pi}+\sum_{j\neq i}\tilde{A}_jm_{Pji}\right)\left(\tilde{a}_iL_{Pi}+\sum_{j\neq i}\tilde{a}_jm_{Pji}\right)}\end{aligned} \qquad \text{S4.8}$$

Like S4.5, this differential is never positive, implying that negative dFDS on the *AVR* gene is generated by migration of the parasite between populations in which the cost of virulence (*b*) or the cost to being unable to infect a *RES* host (*c*) varies between at least some populations.

Section 5: Conditions for stability of the interior equilibrium point

Following SI3, the other selective coefficients of the Jacobian matrix are given by equations S5.1 and S5.2.

$$\frac{d\Delta\rho_i}{d\alpha_i} = \frac{d\Delta\rho_i}{dA_i}\cdot\frac{dA_i}{d\alpha_i} = A_i a_i \left\{ \frac{\tilde{R}_i s_i (1-u_i) L_{Hi}}{\tilde{R}_i L_{Hi} + \sum_{j\neq i} \tilde{R}_j m_{Hji}} \right\} \qquad \text{S5.1}$$

This coefficient (S5.1) is positive by definition of the parameters and allele frequencies. It is the first component of indirect FDS, by which selection for *RES* is enhanced by increasing the *AVR* allele frequency. If the migration rates *m* decrease, selection in deme *i* depends more greatly on the strength of selection in this deme. On the other hand, increasing m_H enhances the influence of the other *n-1* demes on selection in deme *i*. This implies that if selection for *RES* in deme *i* is small (s_i small) but high in other demes (s_j high), low migration rates reduce indirect FDS and thus promote stability. By contrast, increasing migration rates tend to enhance the influence of demes with high indirect FDS, creating more instability.

$$\frac{d\Delta\alpha_i}{d\rho_i} = \frac{d\Delta\alpha_i}{dR_i}\cdot\frac{d\Delta R_i}{d\rho_i} = R_i r_i \left\{ \frac{-c_i A_i L_{Pi}}{\tilde{A}_i L_{Pi} + \sum_{j\neq i} \tilde{A}_j m_{Pji}} \right\} \qquad \text{S5.2}$$

This coefficient (S5.2) is negative. This is also a component of indirect FDS, by which the rate of selection for *AVR* parasites is negatively correlated with increase of *RES* hosts. When the frequency of *RES* plants increases, there is selection of *avr* parasites and therefore negative selection on *AVR*. Increasing migration rates among demes reduce the selection rate against *AVR* parasites. However, this effect depends on *AVR* frequencies in other demes.

The stability of the polymorphic equilibrium point in a deme *i* is given by the eigenvalues of the Jacobian matrix J_i:

$$J_i = \begin{pmatrix} \frac{d\Delta\rho_i}{d\rho_i} & \frac{d\Delta\rho_i}{d\alpha_i} \\ \frac{d\Delta\alpha_i}{d\rho_i} & \frac{d\Delta\alpha_i}{d\alpha_i} \end{pmatrix}$$

Stability thus depends in a complex manner on the magnitude of coefficients of direct FDS (S4.5and S4.8) but also on indirect FDS (S5.1 and S5.2). We cannot derive analytically the conditions for the equilibrium points to be stable, but we can analyse how these coefficients change as a function of the parameters of each deme in the meta-population.

Section 6: Frequency of oscillations of the simple gene-for-gene system close to the unstable interior, non-trivial equilibrium.

The recurrence equations for the simple gene-for-gene model in a single population are linearised around the interior equilibrium point:

$$R' = \hat{R} + \delta R$$
$$A' = \hat{A} + \delta A \qquad \text{S6.1}$$

Substituting S6.1 into the recurrence equation for R (eq. 4 in paper),

$$\frac{\hat{R}+\delta R'}{\hat{r}-\delta R'} = \frac{\hat{R}+\delta R}{\hat{r}-\delta R} \cdot \frac{(1-u)\left\{1-s+s\left(\hat{A}+\delta A\right)\right\}}{1-s} \qquad \text{S6.2}$$

Substituting $\hat{A} = \frac{u(1-s)}{s(1-u)}$ (eq. 5 in paper) into the right-hand part of S6.2,

$$\frac{(1-u)\left\{1-s+s\left(\hat{A}+\delta A\right)\right\}}{1-s} = 1 + \frac{(1-u)s\delta A}{1-s} \qquad \text{S6.3}$$

Substituting S6.3 into S6.2 and letting $\tilde{s} = (1-u)s/(1-s)$,

$$\frac{\hat{R}+\delta R'}{\hat{r}-\delta R'} = \frac{\hat{R}+\delta R}{\hat{r}-\delta R} \cdot (1+\tilde{s}\delta A) \qquad \text{S6.4}$$

As $\delta R \delta R'$ is negligible compared to $\hat{R}\hat{r}$ close to the interior equilibrium point, S6.4 simplifies to

$$\delta R' \cong \delta R + \left(\hat{R}\hat{r} - \hat{R}\delta R' + \hat{r}\delta R\right) \cdot \tilde{s}\delta A \qquad \text{S6.5}$$

S6.5 can be solved if the system is very close to equilibrium such that δA, δR and $\delta R'$ are all so small (e.g. <0.01) that all higher powers are negligible. In this case,

$$\delta R' = \delta R + \hat{R}\hat{r}\tilde{s}\delta A \qquad \text{S6.6}$$

Similarly,

$$\delta A' = \delta A - \hat{A}\hat{a}\tilde{c}\delta R \qquad \text{S6.7}$$

where $\tilde{c} = c/(1-b)$. In matrix form,

$$\begin{pmatrix} \delta R' \\ \delta A' \end{pmatrix} = \begin{pmatrix} 1 & \hat{R}\hat{r}\tilde{s} \\ -\hat{A}\hat{a}\tilde{c} & 1 \end{pmatrix} \begin{pmatrix} \delta R \\ \delta A \end{pmatrix} \qquad \text{S6.8}$$

The eigenvalues (λ) of the system are the solutions of

$$(1-\lambda)^2 + \varepsilon = 0 \qquad \text{S6.9}$$

where

$$\varepsilon = \hat{R}\hat{r}\hat{A}\hat{a}sc = \frac{b(c-b)u(s-u)}{(1-b)c(1-u)s} \qquad \text{S6.10}$$
$$= \hat{R}\hat{a}u \text{ if } c=1$$

Solving S6.9 for λ,

$$\lambda = 1 \pm i\sqrt{\varepsilon} \qquad \text{S6.11}$$

The frequency (θ) of the oscillations is thus (Goldberg 1986)

$$\theta \approx (2\pi)^{-1} \text{arc cos } (1+\varepsilon)^{-½}$$
$$= \frac{1}{2\pi}\arccos\left\{1+\frac{b(c-b)u(s-u)}{(1-b)c(1-u)s}\right\}^{-0.5} \qquad \text{S6.12}$$

When $s > u$ the frequency of oscillations depends generally weakly on s, and is therefore mainly a function of u and b (S6.12). Note that cycling behaviour does not occur if $s<u$ because the result of S6.12 is then complex.

For simplicity, we assume c=1 (Tellier & Brown 2007a,b) in our simulations, because in many plant diseases, *AVR* pathogens infect *RES* plants weakly if at all, so

$$\theta \approx \frac{1}{2\pi}\arccos\left\{1+\frac{bu(s-u)}{s(1-u)}\right\}^{-0.5} \qquad \text{S6.13}$$

Section 7: The strength of direct frequency-dependent selection depends on the difference of oscillation frequencies in a two-deme meta-population

As demonstrated in Section 6, the frequency of oscillations in a given deme can be approximated around the equilibrium point, without migration by:

$$\theta \approx (2\pi)^{-1} \arccos\left\{1+\hat{R}\hat{a}u\right\}^{-0.5} \quad \text{S7.1}$$

(S6.11). So if two demes 1 and 2 have different environments, the difference of their frequencies of oscillation $\Delta\theta = \theta_1 - \theta_2$ is:

$$\Delta\theta \approx (2\pi)^{-1}\left[\arccos\left\{1+\hat{R}_1\hat{a}_1u_1\right\}^{-0.5} - \arccos\left\{1+\hat{R}_2\hat{a}_2u_2\right\}^{-0.5}\right] \quad \text{S7.2}$$

(S6.13). Since *Rau* is much less than 1 for reasonable values of u, b and s, a series of approximations leads to

$$\Delta\theta \approx (4\pi)^{-1}\left|\hat{R}_1\hat{a}_1u_1 - \hat{R}_2\hat{a}_2u_2\right| \quad \text{S7.3}$$

Replacing the equilibrium frequencies by their values from Eq. 5 in the text,

$$\Delta\theta \approx \frac{1}{4\pi}\left|\frac{b_1u_1(s_1-u_1)}{s_1(1-u_1)} - \frac{b_2u_2(s_2-u_2)}{s_2(1-u_2)}\right| \quad \text{S7.4}$$

$\Delta\theta$ is thus a monotonic increasing function of $|b_1 - b_2|$ and $|s_1 - s_2|$, and reaches a maximum with respect to $|u_1 - u_2|$ when $u_i \lesssim s_i / 2$

Direct FDS is affected in the same way as $\Delta\theta$ by differences in the values of b, u and s between demes, becoming larger, with more negative values of $d\Delta\rho/dp$ and $d\Delta\alpha/d\alpha$ as the difference between the costs in each deme increases, so long as $u_i \lesssim s_i / 2$.

We present an analysis of the case with equal costs of resistance (u) and disease (s) in each deme, so $\hat{A}_1 = \hat{A}_2 = \hat{A}$ (eq. 5 in paper), and no pathogen migration (m_P=0), so $\hat{R}_i = b_i$ (S3.12). Let b_2=b and b_1=b+β, assuming without loss of generality that the cost of virulence is greater in deme 1 (with $u_1 = u_2 = u$ and $s_1 = s_2 = s$). We consider the value of direct FDS when all allele frequencies are at equilibrium. Substituting these values into the equation for dFDS on resistance (S4.5) and simplifying for two populations leads to

$$\frac{d\Delta\rho_1}{d\rho_1} = -\frac{\tilde{R}_1(b+\beta)\tilde{r}_2m_HL_H + \tilde{r}_1(1-b-\beta)\tilde{R}_2m_HL_H + \tilde{R}_2\tilde{r}_2{m_H}^2}{\left(\tilde{R}_1L_H + \tilde{R}_2m_H\right)\left(\tilde{r}_1L_H + \tilde{r}_2m_H\right)} \quad \text{S7.5}$$

where $L_H = 1 - m_H$. As the mean fitness of the host, $\bar{w}_H$, is $1-s$ when $R = \hat{R}$ and $A = \hat{A}$ and s is the same in both demes,

$$\frac{d\Delta\rho_1}{d\rho_1} = -m_H \frac{(b+\beta)^2(1-b)(1-m_H)+(1-b-\beta)^2 b(1-m_H)+b(1-b)m_H}{\{b+\beta(1-m_H)\}\{1-b-\beta(1-m_H)\}} \quad \text{S7.6}$$

Assuming that β is small enough for β^2 to be negligible compared to b, S7.6 simplifies to:

$$\frac{d\Delta\rho_1}{d\rho_1} \approx \frac{-b(1-b)m_H}{\beta(1-2b)(1-m_H)+b(1-b)} \quad \text{S7.7}$$

Hence $d\Delta\rho_1/d\rho_1 < 0$ under the conditions stated, and substituting S7.3 (with the simplifications described above S7.5) into S7.7,

$$\frac{d\Delta\rho_1}{d\rho_1} \approx \frac{-b(1-b)u\hat{a}m_H}{4\pi\Delta\theta(1-2b)(1-m_H)+b(1-b)u\hat{a}} \quad \text{S7.8}$$

This implies that as the natural oscillation frequencies in the two demes diverge ($\Delta\theta$ increases as β increases) because the costs of virulence b_1 and b_2 differ between demes, the strength of direct FDS on R changes in the two demes. With increasing $\Delta\theta$ (13, 15), direct FDS becomes more negative in the deme with the lower cost b (here deme 2) , with selection against resistance accelerating more steeply with increasing frequency of resistance, and less negative in the deme with the higher cost b (here deme 1).

Similarly, when there are equal costs of virulence in each deme (b_1=b_2=b) and there is no host migration (m_H=0), we assume different costs of virulence u ($u_2 = u$ and $u_1 = u + \varepsilon$ with $s_1 = s_2 = s$) or of disease s ($s_1 = s$ and $s_2 = s + \sigma$ with $u_1 = u_2 = u$) between demes. This translates into either $\hat{A}_1 = \hat{A} + \varepsilon$ and $\hat{A}_2 = \hat{A}$, such that ε^2 is negligible with respect to $\hat{A}$ or $\hat{A}_1 = \hat{A}$ and $\hat{A}_2 = \hat{A} + \sigma$, provided that σ^2 is negligible with respect to $\hat{A}$. Noting that the mean fitness of the pathogen at equilibrium is $1-b$,

$$\frac{d\Delta\alpha_1}{d\alpha_1} \approx \frac{-\hat{A}\hat{a}m_P}{\varepsilon(\hat{a}-\hat{A})(1-m_H)+\hat{A}\hat{a}} \quad \text{S7.9}$$

Or

$$\frac{d\Delta\alpha_1}{d\alpha_1} \approx \frac{-\hat{A}\hat{a}m_P}{\sigma(\hat{a}-\hat{A})(1-m_H)+\hat{A}\hat{a}}$$

Substituting S7.4 into S7.9,

$$\frac{d\Delta\alpha_1}{d\alpha_1} \approx \frac{-bu\hat{A}\hat{a}m_P}{4\pi\Delta\theta(\hat{a}-\hat{A})(1-m_H)+bu\hat{A}\hat{a}} \quad \text{S7.10}$$

Since u in Eq. S7.10 is a constant, the effect of differences between demes in the cost of resistance is expressed through $\Delta\theta$. Providing that $u_i \lesssim s_i / 2$ (S7.4), as s or u diverges between demes, $\Delta\theta$ increases (S7.4, because ε or σ increase), direct FDS alters, with $d\Delta\alpha_2/d\alpha_2$

becoming more negative in the deme with lower u or higher s (here deme 2). Conversely, $d\Delta\alpha_1/d\alpha_1$ becomes less negative in the deme with the higher u or lower s (here deme 1).

Section 8: The special case of a homogeneous environment

In a homogeneous environment the characteristics of co-evolution are identical among all demes, thus for any deme i, $u_i=u$, $s_i=s$ and $b_i=b$. The equilibrium state for plant and parasite allele frequencies is also identical in all demes.

$$R_i = \widehat{R} = b \text{ and } \widehat{A_i} = \widehat{A} = u(1-s)/s(1-u)\ \forall i \qquad \text{S8.1}$$

Substituting S8.1 into S4.5 and S4.8, $d\Delta\rho_i/\partial\rho_i = d\Delta\alpha_i/\partial\alpha_i = 0$, meaning that the non-trivial equilibrium point is locally always unstable. However, numerical simulations show that when demes have very different initial frequencies, gene frequencies may converge towards this equilibrium point (Figure 3 in text). After a few generations, however, the demes become synchronised again and then show unstable behaviour.

Assume that the initial frequencies of *RES* are different between demes, with $R_i = \widehat{R} + x_i$ (and $r_i = \hat{r} - x_i$)

$$\frac{R_i'}{r_i'} = \frac{\left(\widehat{R}+x_i\right)(1-u_i)(1-s_i a_i)L_{Hi} + \sum_{j\neq i}\left(\widehat{R}+x_j\right)\left(1-u_j\right)\left(1-s_j a_j\right)m_{Hji}}{\left(\hat{r}-x_i\right)(1-s_i)L_{Hi} + \sum_{j\neq i}\left(\hat{r}-x_j\right)\left(1-s_j\right)m_{Hji}} \qquad \text{S8.2}$$

For simplicity, we derive $d\Delta\rho_i/\partial\rho_i$ in a system with two demes. In deme 1, S8.2 becomes:

$$\frac{R_1'}{r_1'} = \frac{\left\{\widehat{R}+x_1+m_{Hj}\left(x_2-x_1\right)\right\}(1-u)(1-sa)}{\left\{\hat{r}_1+x_1+m_{Hj}\left(x_2-x_1\right)\right\}(1-s)} \qquad \text{S8.3}$$

Hence

$$\frac{d\Delta\rho_1}{d\rho_1} = \frac{-m_{Hj}\left(x_2-x_1\right)(1-2b)}{\hat{r}\widehat{R}+m_H\left(x_2-x_1\right)(1-2b)} \qquad \text{S8.4}$$

This differential is negative, implying the existence of negative direct FDS, so long as $m_H\left(x_1-x_2\right) < \hat{r}\widehat{R}$ and b is small.

Interestingly, when the initial host gene frequencies are distant from the equilibrium frequency, *i.e.* when x_1, $x_2 > 0$, there is direct FDS because $d\Delta\rho_1/\partial\rho_1 < 0$ (likewise for the parasite). This means that in one or two demes, gene frequencies can start to spiral towards the equilibrium point. However, as R_1 approaches equilibrium, x_1 and x_2 diminish to become negligible in the vicinity of the equilibrium point ($x_1 \approx x_2 \approx 0$). Note that $d\Delta\rho_1/\partial\rho_1$ is more negative when the migration rate is high so values of x_i decrease more rapidly. As a result, when close to the equilibrium point, $x_1 \approx x_2 \approx 0$ the system collapses to the always unstable model described in the text and $d\Delta\rho_1/\partial\rho_1 = 0$. Then frequency dynamics in the two demes become synchronised again (Figure 3 in the text). A similar analysis shows that equivalent results apply to the parasite's gene frequencies.

References cited in the Additional File 1

Bulmer, M. G. 1972 Multiple niche polymorphism. *The American Naturalist* **106**, 254-257.

Gliddon, C. & Strobeck, C. 1975 Necessary and sufficient conditions for multiple-niche polymorphism in haploids. *The American Naturalist* **109**, 233-235.

Goldberg, S. 1986 *Introduction to difference equations*. Mineola, USA: Dover Publications.

Karlin, S. & Campbell, R. B. 1980 Selection-migration regimes characterized by a globally stable equilibrium. *Genetics* **94**, 1065-1084.

Maynard-Smith, J. 1970 Genetic polymorphism in a varied environment. *The American Naturalist* **104**, 487-490.

Nagylaki, T. 1992 *Introduction to Theoretical Population Genetics*. Biomathematics Texts. Berlin-Heidelberg-New York: Springer-Verlag.

Nagylaki, T. & Lou, Y. 2006 Evolution under the multiallelic Levene model. *Theoretical Population Biology* **70**, 401-411.

Nagylaki, T. & Lou, Y. 2007 Evolution under multiallelic migration-selection models. *Theoretical Population Biology* **72**, 21-40.

Prout, T. 1968 Sufficient Conditions for Multiple Niche Polymorphism. *American Naturalist* **102**, 493-496.

Star, B., Stoffels, R. J. & Spencer, H. G. 2007a Evolution of fitnesses and allele frequencies in a population with spatially heterogeneous selection pressures. *Genetics* **177**, 1743-1751.

Star, B., Stoffels, R. J. & Spencer, H. G. 2007b Single-locus polymorphism in a heterogeneous two-deme model. *Genetics* **176**, 1625-1633.

Strobeck, C. 1974 Sufficient conditions for polymorphism with n niches and m mating groups. *The American Naturalist* **108**, 152-156.

Tellier, A. & Brown, J. K. M. 2007a Polymorphism in multilocus host-parasite coevolutionary interactions. *Genetics* **177**, 1777-1790.

Tellier, A. & Brown, J. K. M. 2007b Stability of genetic polymorphism in host-parasite interactions. *Proceedings of the Royal Society B-Biological Sciences* **274**, 809-817.
