# Supplementary material for: Spatial heterogeneity, frequency-dependent selection and polymorphism in host-parasite interactions
Source: BMC Evol Biol. 2011 Nov 1;11:319. doi: 10.1186/1471-2148-11-319 (PMC3273489; doi:10.1186/1471-2148-11-319)

Additional File 2

Figure S1: Dynamics of allele frequencies in a two-deme model with equal parameter values between demes (for simplicity, c=1). Initial allele frequencies are different for deme 1 (R,a)=(0.001,0.5) and for deme 2 (0.15,0.5), (u_1= u_2 =0.05, b_1= b_2 =0.05, s_1= s_2 =0.1, m=0.03): initially, inward transient spiralling toward equilibrium, followed by synchronisation of allele frequencies between demes, then outward spiralling away from the interior equilibrium.

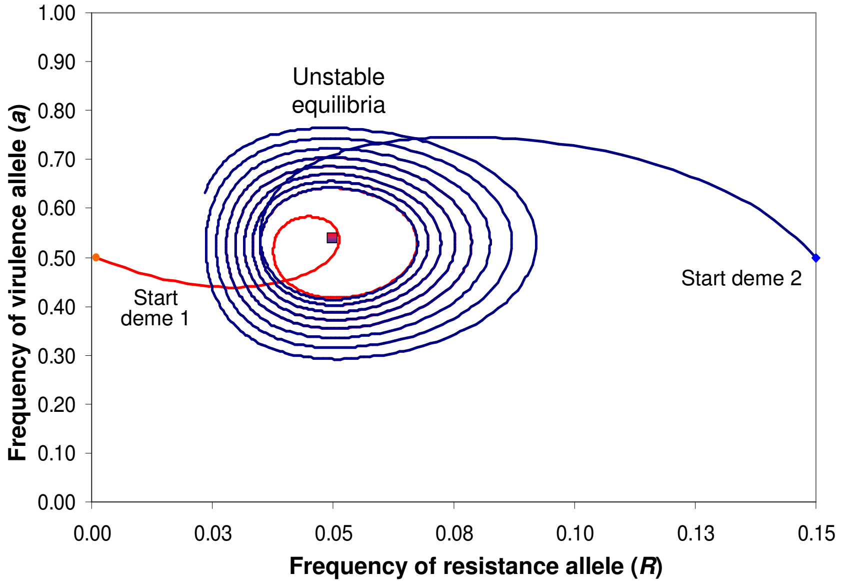

Supplement: Additional file 2 — Figure S1. The file contains the Figure S1. [file 1471-2148-11-319-S2.PDF]
